# Sensitive Assay for the Lactonase Activity of Serum Paraoxonase 1 (PON1) by Harnessing the Fluorescence Turn-On Characteristics of Bioorthogonally Synthesized and Geometrically Controlled Chemical Probes

**DOI:** 10.3390/molecules27082435

**Published:** 2022-04-09

**Authors:** Bo-Kai Fang, Chia-Yen Dai, Scott Severance, Chi-Ching Hwang, Chien-Hui Huang, Sin-Yu Hou, Bao-Lin Yeh, Ming-Mao Gong, Yun-Hao Chou, Jeh-Jeng Wang, Tzu-Pin Wang

**Affiliations:** 1 Department of Medicinal and Applied Chemistry, Kaohsiung Medical University, Kaohsiung 80708, Taiwan; fangken880705@gmail.com (B.-K.F.); jennyhuang4128@gmail.com (C.-H.H.); sinyu95017@gmail.com (S.-Y.H.); p5012830@gmail.com (B.-L.Y.); orange110129@gmail.com (M.-M.G.); jjwang@kmu.edu.tw (J.-J.W.); 2 School of Medicine, College of Medicine, Kaohsiung Medical University, Kaohsiung 80708, Taiwan; d820195@kmu.edu.tw; 3 Department of Internal Medicine, Kaohsiung Medical University Hospital, Kaohsiung Medical University, Kaohsiung 80708, Taiwan; 4 College of Professional Studies, National Pingtung University of Science and Technology, Pingtung 91201, Taiwan; 5 Department of Molecular and Cellular Sciences, Liberty University College of Osteopathic Medicine, Lynchburg, VA 24515, USA; smseverance@liberty.edu; 6 Department of Biochemistry, Faculty of Medicine, College of Medicine, Kaohsiung Medical University, Kaohsiung 80708, Taiwan; cchwang@kmu.edu.tw (C.-C.H.); s1235740@gmail.com (Y.-H.C.); 7 Department of Medical Research, Kaohsiung Medical University Hospital, Kaohsiung Medical University, Kaohsiung 80708, Taiwan

**Keywords:** fluorescence turn-on, chemical probe, bicyclononyne, paraoxonase 1, lactonase

## Abstract

The lactonase activity of paraoxonase 1 (PON1) has a crucial antiatherogenic function, and also serves as an important biochemical marker in human blood because the aberrant lactonase activity of PON1 is a key indicator for a number of diverse human diseases. However, no sensitive fluorescence assays that detect PON1 lactonase activity are available. We report the synthesis of two fluorescence turn-on chemical probes **16a** and **16b** (**16**) able to quantify PON1 lactonase activity. The chemical probes were constructed utilizing a disulfide-containing bicyclononyne, derivatives of rhodamine B and carboxyfluorescein, and reactions including copper-free azide–alkyne cycloaddition. Fluorescence quenching in **16** was characterized by spectroscopic studies and was mainly attributed to the effect of contact quenching. Kinetic analysis of **16b** confirmed the outstanding reactivity and specificity of **16b** with thiols in the presence of general base catalysts. The **16b**-based assay was employed to determine PON1 lactonase activity, with a linear range of 10.8–232.1 U L^−1^ and detection limit (LOD) of 10.8 U L^−1^, to quantify serum PON1 activity in human sera, and to determine the *K*_i_ of 20.9 μM for the 2-hydroxyquinoline inhibition of PON1 lactonase. We are employing **16b** to develop high-throughput assays for PON1 lactonase activity.

## 1. Introduction

Many human diseases, including cardiovascular and neurodegenerative diseases, cancer, obesity, type 2 diabetes mellitus, aging, Crohn’s disease, microbial infections, and non-alcoholic fatty liver disease, are characterized by chronic or acute oxidative stress and exacerbated inflammation in patients [1,2,3,4]. The detrimental disease states can be partly averted by the normal functions of three proteins—PON1, PON2 and PON3—in the paraoxonase (PON) family, which play anti-oxidative and anti-inflammatory roles in safeguarding human health [1,5]. These proteins have potential as biomarkers or therapeutic targets for managing certain human disorders [2,3,5,6,7,8,9,10]. For example, the interplay of PON1 (EC 3.1.8.1), the most studied member of the PON family, with high-density lipoproteins (HDL) potentiates the critical antioxidant properties of low-density lipoproteins (LDL) and bestows the antiatherogenic effects of PON1 [11,12,13,14,15]. In humans, PON1 is expressed mainly in the liver, and is secreted into the bloodstream where PON1 interacts with HDL, which shelters the hydrophobic *N*-terminal region of PON1 in aqueous solution [16]. The formation of the PON1–HDL complex is essential for PON1 to engage in catalysis with its substrates [15] and the degradation of lipid peroxides in HDL and LDL [17,18]. Human PON1 is a calcium-dependent glycoenzyme with an approximate molecular mass of 43 kDa, and is composed of 354 amino acids that form a six-bladed β-propeller structure with four β strands in each blade [16,19]. Early studies on PON1-catalyzed hydrolysis of paraoxon—the toxic metabolite of the organophosphorous ester insecticide parathion—led to the names of the PON proteins [12,13]. Nevertheless, PON1 is actually a promiscuous enzyme, because, besides paraoxonase activity (i.e., phosphotriesterase activity), it has lactonase and ester hydrolase activities shown to hydrolyze thiolactones, unsaturated aliphatic esters, aromatic carboxylic esters, and carbamates [14,15,16,20]. In addition, the physiological role of PON1 has been confirmed to be as a lactonase enzyme with lactones as the physiological substrate [21]. Moreover, PON1 is able to hydrolyze homocysteine thiolactone, a cytotoxic metabolite of the pro-oxidant homocysteine [13,22]. Because PON1 could play a critical role as an antioxidant enzyme and a promoter of lipid and homocysteine homeostasis in the antioxidant system by removing toxic homocysteine thiolactone and oxidized phospholipids from circulation, a decrease in blood PON1 levels and lactonase activity has been associated with diseases involving elevated oxidative stress and inflammation [1,3,10,23,24]. Sensitive PON1 assays for the detection of abnormal lactonase activity in the blood are indispensable to the diagnosis and treatment of life-threatening diseases. Consequently, there is an urgent demand for straightforward and accurate techniques capable of sensitively quantifying the lactonase activity of PON1 in the blood.

Intriguingly, a limited number of methods have been developed to measure PON1 activity in samples, and most of them are able to determine only the esterase and/or phosphotriesterase activity of PON1. A few zymographic [25], HPLC [20,26,27] and mass spectrometric [28] detection methods have been reported for quantification of PON1 activity. Yet, PON1 activity is typically determined by spectroscopic methods based on UV-Vis absorption [12,21,29,30,31,32,33], chemiluminescence [34] and fluorescence [30,35,36,37] measurements. The UV-Vis-based colorimetric assays take advantage of the ability of PON1 to hydrolyze substrates, and they are currently more widely used, probably due to their lower cost and broader availability. However, the bioanalytical applications of the UV-Vis absorption methods for PON1 activity are compromised by the intrinsically low sensitivity of UV-Vis spectrophotometry, and by significant interference from naturally occurring molecules in the blood. In contrast, fluorometric analysis has the potential to improve sensitivity and to lower interference in PON1 activity assays. A few chemilumigenic and fluorogenic methods have been employed to measure PON1 activity in standard buffer solutions and blood samples [30,34,35,36,37]. Unfortunately, the fluorometric methods for PON1 activity analysis are currently plagued with the use of highly toxic reactants, low sensitivity, and, most importantly, the inability to determine the lactonase activity of PON1. There is a need for a straightforward and selective PON1 lactonase assay based on novel and benign fluorescence reagents. We were thus motivated to harness biocompatible and bioorthogonal chemistry, which we have previously utilized [38] to develop sensitive fluorescent chemical probes capable of specifically quantifying PON1 lactonase activity in human blood samples.

**Scheme 1:**
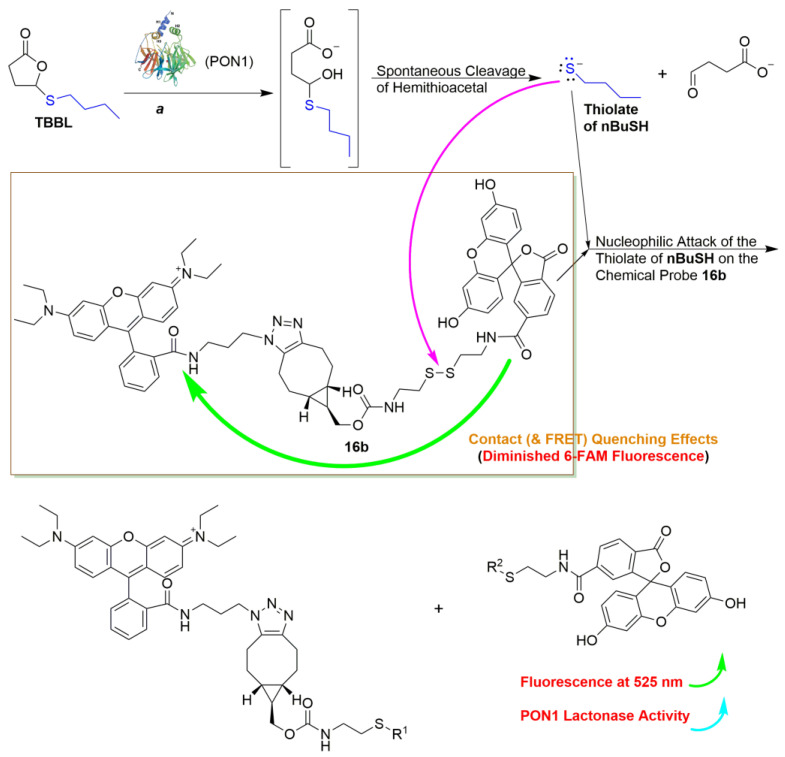
Use of the chemical probe **16b** to quantitatively analyze catalysis and inhibition of PON1 lactonase activity. The PON1-catalyzed hydrolysis of thiobutyl butyrolactone (TBBL, a thiolactone) would provide a labile hemithioacetal, which spontaneously cleaves to give 1-butanethiol (nBuSH) as one of the products. The thiolate of nBuSH is highlighted in blue. Subsequently, the thiolate nucleophilically attacked (the pink arrow) the disulfide bond in **16b** to obliterate the fluorescence quenching effects (the green arrow) and to liberate the 6-carboxyfluorescein (6-FAM) fluorescence. Increments in the 6-FAM fluorescence (the light green arrow) are, therefore, proportional to increases in PON1 lactonase activity (the light blue arrow). ***a***: (TBBL), Tris, Ca^2+^, pH 8.0. R^1^ and R^2^, H or the thiolate of nBuSH.

Here, we report the successful synthesis of the novel fluorescence turn-on chemical probes **16a** and **16b** (**16**), capable of accurately quantifying PON1 lactonase activity. We have demonstrated that the **16b**-based fluorescence assay is the first of its kind able to directly quantify PON1 lactonase activity in samples (Scheme 1), provide a broad linear detection range of PON1 lactonase activity with low limit of detection (LOD), and accurately determine PON1 lactonase activity in serum from humans. The chemical probe **16b** will be an invaluable reagent to meet the pressing need for the sensitive and rapid quantification of PON1 lactonase activity in serum in clinical settings.

## 2. Results and Discussion

### 2.1. Synthesis of the Fluorescent Chemical Probes ***16b***

Analogous to a recently reported fluorescence chemical probe for the quantification of butyrylcholinesterase (BChE) activity [38], the fluorescent chemical probes **16b** was also synthesized from ***exo*-6** (Scheme S1, Supplementary Materials, SM), a crucial disulfide-containing bicyclononyne capable of performing the important bioorthogonal and biocompatible copper-free strained-promoted azide-alkyne cycloaddition (SPAAC) reactions [39] for the effective synthesis of **16b**. We previously indicated that the trifunctional nature of ***exo*-6** provides the appropriate molecule platform to modulate the incorporation of specific fluorophores into one of the two termini in ***exo*-6**, and to facilitate the tuning of the fluorogenic properties of afforded chemical probes [38]. We employed the rhodamine B-carboxyfluorescein (FAM) pair in this current study because the degree of proximity between the fluorophores in the chemical probes could be harnessed to develop a sensitive FAM-based PON1 lactonase activity assay at alkaline pH.

**Scheme 2:**
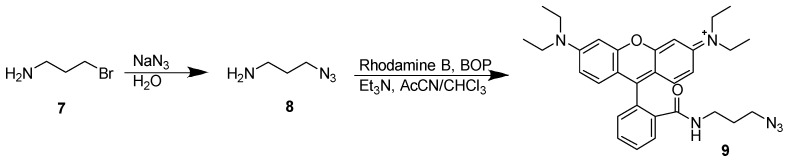
Synthesis of azido-rhodamine B (**9**).

**Scheme 3:**
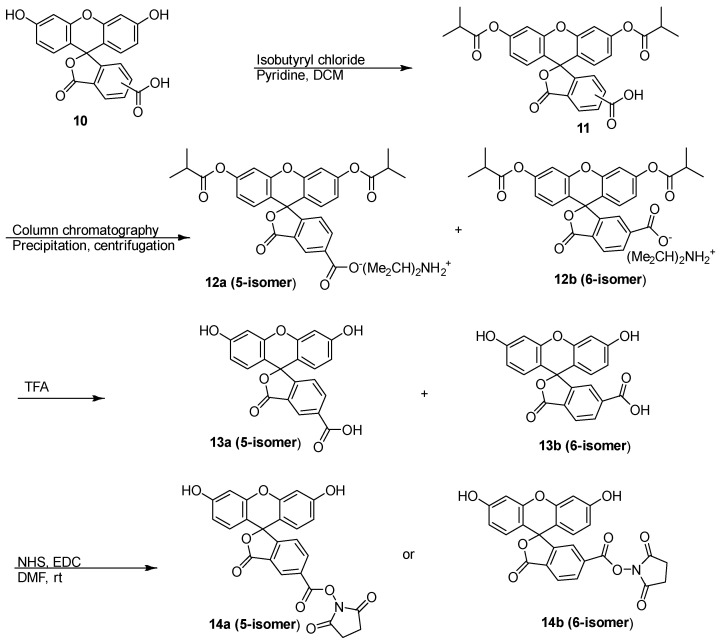
Reaction sequence for separating 5(6)-carboxyfluorescein (FAM) into geometric isomer-pure 5-carboxyfluorescein (5-FAM, **13a**) and 6-FAM (**13b**), which were further derivatized to provide the corresponding NHS esters 14a and 14b, respectively.

Both rhodamine B and FAM were modified to introduce azido and NHS ester groups, respectively, before coupling with ***exo*-6** and achieving synthesis of **16**. For example, the azido derivative of rhodamine B (**9**) was synthesized from the amidation reaction of rhodamine B with **8** (Scheme 2). In addition, we developed an efficient method to effectively separate and purify both 5-carboxyfluorescein (5-FAM, **13a**) and its geometric isomer 6-carboxyfluorescein (6-FAM, **13b**) from the 5(6)-FAM (**10**) mixture, and to convert each geometric isomer-pure FAM to the corresponding FAM-NHS ester (**14**) (Scheme 3). As we will reveal later, the acquisition of the geometric isomer-pure FAM and FAM derivatives was essential to better control the fluorogenic property of the chemical probes and develop more sensitive fluorescence assays for the quantification of PON1 lactonase activity.

**Scheme 4:**
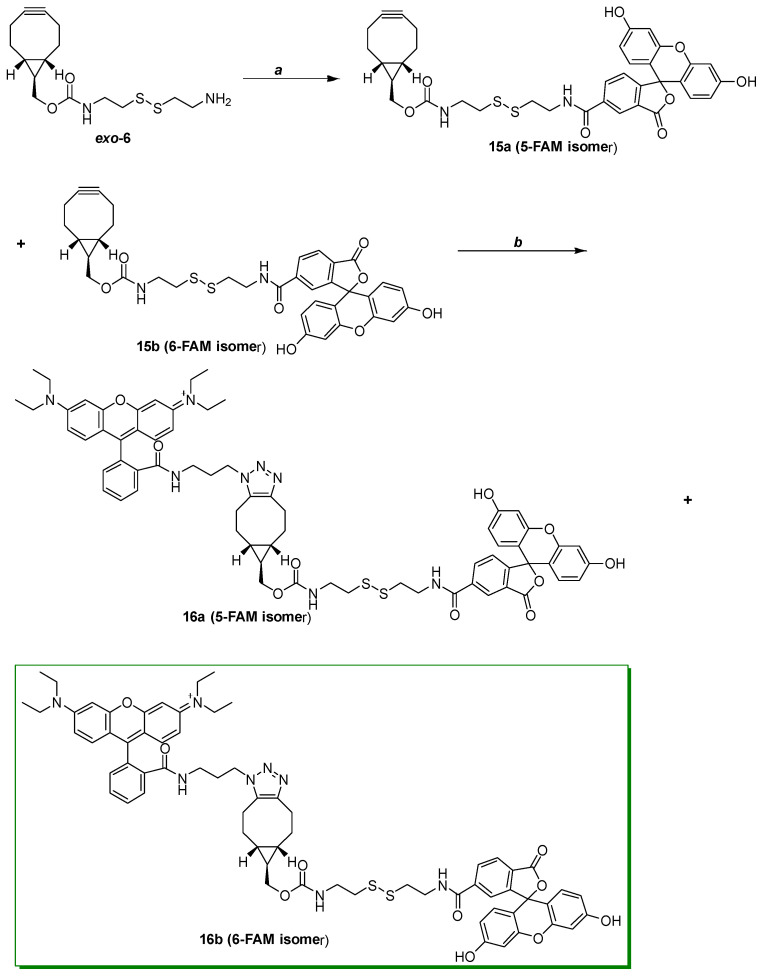
Synthesis of the fluorescence turn-on chemical probes 16 from ***exo*-6**. a: **14a**/**14b**, DIPEA, rt, O/N; b: 9, dioxane, reflux.

Subsequent syntheses of the chemical probes **16a** and **16b**, however, adopted a drastically different approach from that of the BChE chemical probe synthesis [38]. Here, we abandoned the reported one-pot reaction format and employed a two-reaction strategy in order to increase the yields of the chemical probes **16** in the synthesis (Scheme 4). The one-pot reaction used in synthesis of the BChE chemical probe consistently provided a rather poor yield of less than 50% [38]. We reasoned that the low reaction yield problem could be overcome by coupling ***exo*-6** with **14** through a more optimal amidation reaction to give **15**, which was later subjected to a high-yield SPAAC reaction with **9** to give the target chemical probes **16**. Indeed, we successfully synthesized the chemical probes **16** with good yields by following the two-reaction scheme. Specifically, starting from ***exo*-6** and **14a**/**14b**, the combined two-reaction yields are 76.2% for **16a** and 55.1% for **16b**. In addition, the yield difference between **16a** and **16b** suggests that more severe steric hindrance and molecular crowdedness in **14b** and **15b** clearly overwhelmed the accessibility of the 6-carboxylate and the 6-carboxyl NHS ester group in the molecules, compromising the yields of **15b** (61.6%) and **16b** (89.4%), respectively. The apparent disadvantage related to the geometrical constraints of the 6-FAM moiety deteriorating the **16b** synthesis yield, however, endowed **16b** with unique fluorogenic properties useful in developing sensitive assays for PON1 lactonase activity, which will be revealed in the next spectrophotometric studies of the chemical probes.

### 2.2. Contact Quenching and Fluorogenic Properties of the Fluorescent Chemical Probes ***16a*** and ***16b***

**Figure 1:**
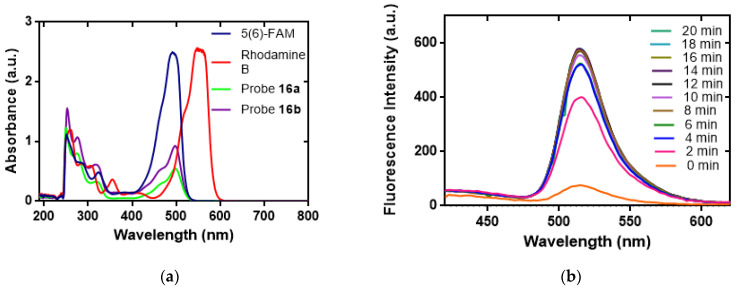
Contact quenching characteristics of the chemical probes **16** revealed by the UV-Vis absorption and the fluorogenic property of one of the chemical probes **16b** when reacting with a thiol. (**a**) The UV-Vis spectra for 50 μM of 5(6)-FAM (the blue curve), rhodamine B (the red curve), and the fluorescence chemical probes **16** (the light green curve for **16a**; the purple curve for **16b**) in phosphate buffer (PB; 10% dimethylformamide (DMF), pH 7.4). (**b**) The time-dependent increase in 6-FAM fluorescence in the reaction of **16b** (1 μM) with L-cysteine (50 mM) in PB.

Contact fluorescence quenching in the chemical probes **16a** and **16b** was characterized by UV-Vis spectrophotometry, and demonstrated the perturbation of the major FAM (λ_max_, ~495 nm) and rhodamine B (λ_max_, ~550 nm) absorption bands in **16** due to the proximity of the fluorophore moieties, which led to the formation of nonfluorescent intramolecular heterodimers in **16** (Figure 1a). The resulting UV-Vis spectra showed the complete disappearance of the characteristic rhodamine B absorption band and the dramatic diminution of the typical FAM absorption band in **16**. Alternatively, the rhodamine B-FAM pair had the ability to perform Förster resonance energy transfer (FRET) and quench the FAM fluorescence in **16**, when maintaining an appropriate distance between the fluorophores. FRET quenching, however, was likely only a minor contributor to the observed diminished FAM fluorescence in **16** (Figure 1a and Figure S1A, SM), because a predominance of FRET effects would require the visible absorption of the fluorophores in **16** in order to stay intact, and to have intensities similar to those of the free fluorophores in the same concentration [40,41,42]. This is obviously not seen in Figure 1a. Indeed, we had to enact a four-fold increase in the concentration of **16** in order to observe rhodamine B’s fluorescence in glacial acetic acid solution (Figure S1B, SM). 

Overall, the results were consistent with past studies demonstrating that a short distance (van der Waals contact) between two fluorophores, or between a fluorophore–quencher pair, in contact quenching constructs would facilitate direct electronic interaction of the fluorophore-excited state with the counterpart chromophore, prohibit the return of the excited fluorophore electrons to the ground state, and be characterized by the significantly altered visible absorption properties of the chromophore pair [38,42,43]. It is noted that the same visible absorption change was documented recently for the BChE chemical probe, which, although it was originally described as a FRET probe, is more appropriately classified as a contact quenching fluorescence chemical probe [38].

**Figure 2:**
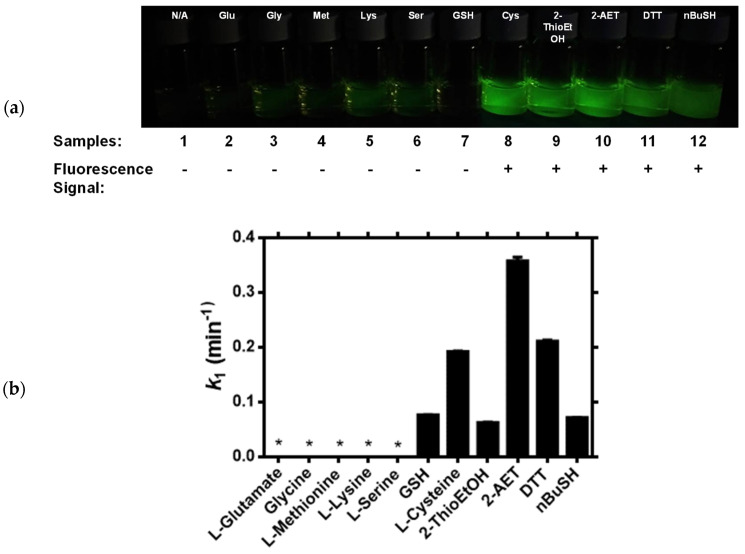
Differential reactivity of **16b** toward reactants as revealed by direct visualization and kinetic analysis. (**a**) Visualization of the 6-FAM fluorescence to characterize the fluorescent emission properties of **16b** in the presence of various reactants. Each vial contained 25 μM of **16b** in the Tris buffer (50 mM Tris, 1 mM Ca^2+^, pH 8.0). In addition, samples **2–12** also included the reactants (50 mM) of L-glutamate, glycine, L-methionine, L-lysine, L-serine, glutathione (GSH), L-cysteine, 2-mercaptoethanol (2-ThioEtOH), 2-aminoethanethiol (2-AET), DL-dithiothreitol (DTT) and 1-butanethiol (nBuSH), respectively. The reactions were carried out in the dark at rt for 1.5 h before the photograph was taken under UV illumination (365 nm). (**b**) Determination of the pseudo-first order rate constant *k*_1_ from each of the reactions of **16b** with various reactants. Please see Section 3.3 for the experimental details. Each reactant was analyzed at least three times in order to acquire the averaged *k*_1_ values and standard deviation (the error bar). The error bars were too short to be visible in some reactions. The symbol * indicates that **16b** had no measurable reactivity, meaning that the values of averaged *k*_1_ and standard deviation could not be determined.

The fluorogenic properties of **16** were confirmed by reacting **16** with L-cysteine in the Tris buffer (Figure 1b and Figure S1A, SM). Significantly, the contact quenching of FAM fluorescence in **16** was quickly and completely obliterated within 15–30 min, indicating that **16** promptly reacted with the thiol-containing L-cysteine. We decided to study the potential use of **16b** as a reagent in the assay used to quantify PON1 lactonase activity, because **16b** was unequivocally a more sensitive and reactive chemical probe that provided fluorogenic properties superior to those of **16a** (Figure 1b and Figure S1A, SM). The fluorescence turn-on properties of **16b** in the presence of various compounds with or without a thiol group (Figure 2a) again confirmed the rapid, thiol-responsive reactivity of **16b,** and the potential of **16b** to be an effective fluorescence chemical probe for the detection of thiols. Clearly, all thiol-containing reactants, including 1-butanethiol (nBuSH, one of the hydrolyzed products of the PON1 substrate 5-(thiobutyl) butyrolactone (TBBL)), were able to swiftly react with **16b** and to increase levels of FAM fluorescence. The discriminative reactivity of **16b** with thiols was also consistent with the pseudo-first order rate constants (*k*_1_) for the reactions (Figure 2b and Figure S2, SM). The qualitative and quantitative measurements of the **16b** reactions demonstrate that, among the studied reactants, 2-aminoethanethiol (2-AET) was the most effective in relieving the contact quenching effect in **16b** and liberating the FAM fluorescence (Figure 2 and Figure S2, SM). We therefore decided to more comprehensively study the fluorogenic mechanism of the reaction between **16b** and 2-AET, because the results would be critical in establishing a **16b**-based assay as a viable method for measuring PON1 lactonase activity.

### 2.3. Mechanistic Studies of the Fluorogenic Reaction of ***16b*** with 2-AET

**Figure 3:**
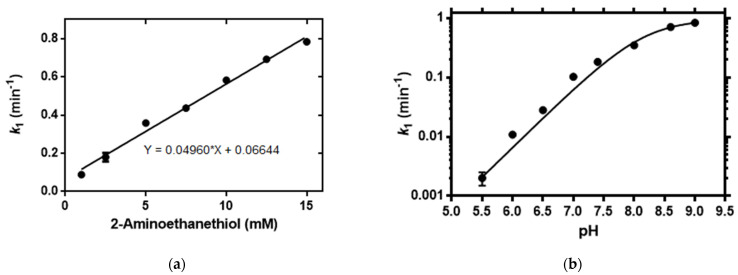
Titration studies to decrypt the reaction mechanism of **16b** with 2-AET. (**a**) Kinetic analysis of 2-AET titration on the 2-AET–**16b** reaction was performed in the presence of 0.4 μM of **16b**. (**b**) Kinetic analysis of pH titration on the 2-AET–**16b** reaction was carried out in the presence of 0.4 μM of **16b** and 5 mM of 2-AET, while varying the pH. Please see Section 3.3 for the experimental details. Each reaction was analyzed at least three times in order to acquire the averaged *k*_1_ values and standard deviation (the error bar).

The kinetic studies of the reaction between a constant **16b** concentration (0.4 μM) and varied 2-AET provide insight into the mechanism of the fluorogenic reaction. We have determined the *k*_1_ values of the reactions of **16b** in the presence of different 2-AET concentrations, and plotted the values of *k*_1_ vs. 2-AET to give a straight line (Figure 3a). Therefore, the 2-AET–**16b** reaction is, overall, second-order, first-order to **16b**, and first-order to 2-AET, with the second-order rate constant *k*_2_ of the reaction equal to 0.83 M^−1^ s^−1^, as determined from the slope of the line. Again, the chemical probe **16b** is similar to the recently reported BChE probe [38]; it supports a S_N_2 nucleophilic substitution reaction mechanism, in which the rapid deprotonation of 2-AET is followed by the rate-limiting nucleophilic attack of the corresponding thiolate on the disulfide bond in **16b** to cleave the disulfide bond and to release the 6-FAM fluorescence.

A survey of pH and metal ion effects on the **16b**–thiol reaction shed further light on the reaction mechanism. We first obtained *k*_1_ for the reactions between 0.4 μM of **16b** and 5 mM of 2-AET in the presence of differing pH and/or buffers. The pH titration results were diagrammed (Figure 3b) and unambiguously show incremental changes in *k*_1_ values, while the pH of the buffers was changed from acidic to alkaline. The values of *k*_1_ approximately plateaued at pH 8.5, which they clearly demonstrated characteristics of general base catalysis to accelerate the 2-AET–**16b** reaction. In addition, the titration study allowed us to determine the p*K*_a1_ of 2-AET to be 8.16 ± 0.07, which is a value consistent with the previously reported 8.19 [44].

Moreover, the potential of metal interference in fluorogenic reactions of **16b** was determined by kinetic studies of reactions in the presence of 0.4 μM of **16b**, 5 mM of L-cysteine, and 1 mM of the studied metal ions (Figure S3A, SM). Only Cu(II) and Fe(III) significantly inhibited the L-cysteine–**16b** reaction. We found that, in the L-cysteine–**16b** reaction containing Mn(II), the cation formed complexes with **16b** and precipitated out of the solution, which rendered kinetic analysis of the reaction impractical. Overall, the second-order thiol–**16b** reaction is characterized by a general base catalysis with a S_N_2 nucleophilic substitution reaction mechanism; moreover, the metal ions prevalent in blood do not interfere in the thiol–**16b** reaction.

Finally, we evaluated the relative importance and role of molecular crowdedness surrounding the disulfide bond in **16b** in bestowing the ability of **16b** to differentially react with various thiol-containing reactants. For example, the *k*_1_ value of the reaction with glutathione (GSH, 0.076 min^−1^) was less than one-fifth of that of the reaction with 2-AET (*k*_1_ of 0.387 min^−1^) (Figure 2b), which suggests that **16b** only moderately reacted with GSH. The previously studied BChE chemical probe also displayed peculiar reactivity toward GSH, and had a significantly diminished fluorogenic property in the presence of GSH [38]. The BChE chemical probe apparently had a unique intrinsic ability to differentiate and rapidly react with most thiol-containing compounds, while remaining rather unreactive to GSH. Subsequent studies have demonstrated that the BChE probe was able to impose steric hindrance on GSH and to prevent the corresponding thiolate of GSH from nucleophilically attacking the disulfide bond in the chemical probe. We thus sought to determine whether the same molecular constraints were also the origin of the distinctive reactivity of **16b** with GSH.

We again exploited two GSH derivatives, a monoethyl ester derivative **17** and a dimethyl ester derivative **18**, both of which were previously synthesized to study the BChE probe (Figure S3B, SM) [38]. Here, **17** and **18** were used as substitutes for GSH in the kinetic analysis of the reactions with **16b**. The chemical probe **16b** reacted with the dimethyl ester of GSH (**18**) similarly to how the BChE probe reacted with **18**. The *k*_1_ value (0.064 min^−1^) of the **18**–**16b** reaction was comparable to that of the GSH–**16b** reaction (0.076 min^−1^; Figure S3C, SM). However, the *k*_1_ (0.038 min^−1^) value of the reaction of **16b** with the monoethyl ester of GSH, i.e., **17**, was half of that for the GSH–**16b** reaction, and was drastically different from that of the reaction of the BChE probe with **17**, which showed completely abolished reactivity and delivered a *k*_1_ essentially equal to zero. Evidently, replacing a methyl group with an ethyl group in GSH enhanced the **16b**-imposed steric obstruction, but was unable to annihilate the reaction with **17**, as was observed in the reaction between the BChE probe and **17**. Thus, GSH’s glycine carboxylate group, which was either ethylated in **17** or methylated in **18**, experienced less constraint caused by **16b** than by the BChE probe. The reduced molecular crowdedness in **16b** might contribute to its improved reactivity with GSH. Consequently, the steric hindrance of **16b** experienced by GSH is still the most plausible explanation for the *k*_1_ value of 0.076 min^−1^ in the GSH–**16b** reaction being smaller than that in the 2-AET–**16b** reaction, but not significantly small enough to dramatically reduce the reaction rate. The same molecular constraints of **16b** might also hinder the reaction with nBuSH, to give a *k*_1_ of 0.072 min^−1^ (Figure 2b), even though the structure of nBuSH is unrelated to that of GSH.

Since GSH is found naturally and abundantly in human blood, with a concentration of 2.09 ± 1.15 μM in the plasma of healthy humans [45], and had reactivity with **16b** comparable to that of nBuSH in the **16b**–nBuSH reaction, it could potentially interfere in a **16b**-based assay used to measure the activity of PON1 lactonase in blood samples. Consequently, as described in Section 3.4, we have developed a strategy to avoid interference from serum GSH, and have successfully exploited **16b** to accurately quantify PON1 lactonase activity in serum.

### 2.4. Development of the ***16b***-Based Fluorescence Turn-On Assay for Measuring PON1 Lactonase Activity

**Figure 4:**
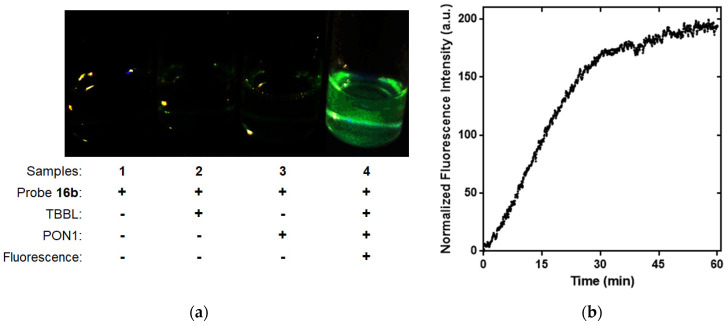
The chemical probe **16b** used for accurate determination of PON1 lactonase activity. (**a**) Each vial contained 2 μM (in dimethyl sulfoxide (DMSO), 0.5%) of **16b** in the Tris buffer, and various combinations of PON1 (7.79 U L^−1^, 50% glycerol) and TBBL (5 mM in acetonitrile (ACN), 0.2%). The reactions were carried out at rt for 15 min and visualized and photographed under UV lamp illumination. (**b**) Catalysis of a PON1-TBBL–**16b** reaction system was monitored spectrofluorometrically; the reaction contained **16b** (0.8 μM in DMSO, 1.6%), rePON1 (232.1 U L^−1^, 50% glycerol), and TBBL (10 mM in ACN, 1%) in the Tris buffer at 25 °C. The normalized FL intensity at 525 nm was acquired by subtracting a background fluorescence of **16b** at the same wavelength from the original fluorescence intensity data.

The fluorescence turn-on assay based on **16b** (Scheme 1) demonstrated an ability to specifically and accurately measure PON1 lactonase activity when it was qualitatively revealed that **16b** reacted only with the catalytic reaction product nBuSH in the presence of TBBL and PON1 (Figure 4a). The quantitative analysis of the PON1-TBBL–**16b** catalysis system also justified the use of **16b** for the accurate detection of PON1 lactonase activity (Figure S4, SM). The time-dependent fluorescence change curve of a PON1-TBBL–**16b** reaction exhibited the classical Michaelis–Menten model of enzyme kinetics, and facilitated the subsequent determination of PON1 lactonase activity in terms of initial velocities, *v*_i_ (Figure 4b). Thus, the chemical probe **16b** has the critical characteristics necessary for the sensitive and specific quantification of the PON1 lactonase activity in human blood samples.

### 2.5. Sensitive and Accurate Determination of PON1 Lactonase Activity in Human Serum by the Fluorescence Turn-On Assay in the Presence of ***16b***

**Figure 5:**
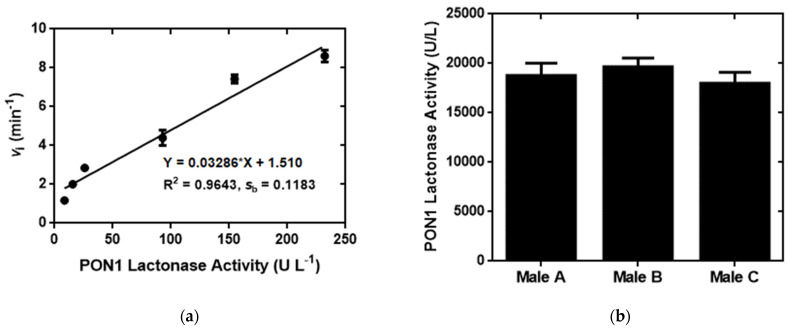
The **16b**-based assay for the determination of PON1 lactonase activity in sera. (**a**) PON1 standards obtained from *E. coli* were analyzed using the **16b**-based method that afforded the linear regression calibration curve of PON1 lactonase activity vs. initial velocity (*v*_i_), and gave an LOD of 10.8 U L^−1^ and a linear detection range of 10.8–232.1 U L^−1^. (**b**) PON1 lactonase activity in serum samples from three healthy males was measured using the **16b**-based assay. Please see Section 3.4 for the experimental details. Each reaction was analyzed at least three times in order to acquire the averaged values and standard deviation (the error bar) of *v*_i_ or PON1 lactonase activity.

The results demonstrating the ability of the **16b**-based assay to accurately quantify PON1 lactonase activity in samples (Figure 4 and Figure S4, SM) encouraged us to employ the nBuSH-responsive, 6-FAM fluorescence-releasing **16b** to determine PON1 lactonase activity in serum. We began with the kinetic analysis of the time-dependent fluorescence turn-on of **16b** in the presence of rePON1 standards with different activities, in order to acquire the corresponding values of *v*_i_. The linear regression analysis of PON1 activity vs. *v*_i_ showed that the **16b**-based assay provided a linear calibration curve with a slope *m* of 0.033 and a good linear detection range of 10.8–232.1 U L^−1^ (Figure 5a). The LOD of PON1 lactonase activity determined by the **16b**-based assay was calculated to be 10.8 U L^−1^, according to the equation of LOD = 3*s*_b_/*m*, in which the standard deviation *s*_b_ of 0.118 was determined from three blank reactions. The quantitative method based on **16b** is the first fluorescence turn-on assay for the sensitive and accurate measurement of PON1 lactonase activity, and is characterized by a linear detection range of 10.8–232.1 U L^−1^ and an LOD of 10.8 U L^−1^.

The calibration curve in Figure 5a was employed to determine PON1 lactonase activity in serum samples from three healthy males. The **16b**-based assay successfully quantified PON1 lactonase activity in the serum samples (Figure 5b) and provided the PON1 lactonase activity in the range of 17,800–19,500 U L^−1^. The PON1 lactonase activity in human serum was previously reported to be 3800 ± 1900 U L^−1^, according to a colorimetric Ellman’s assay [33], which is only approximately one-fifth of the value provided by the current study. The disparity between the values of PON1 lactonase activity in human serum could be due to the lower sensitivity of Ellman’s assay in comparison with the current fluorescence turn-on assay based on **16b**. This may suggest that the **16b**-based fluorescence assay is more capable of sensitively and accurately determining PON1 lactonase activity in serum samples.

### 2.6. Kinetic Analysis of PON1 Inhibition to Corroborate the Requirement of PON1 Lactonase Activity for Turning on the 6-FAM Fluorescence in the ***16b***-Based Assay

**Figure 6:**
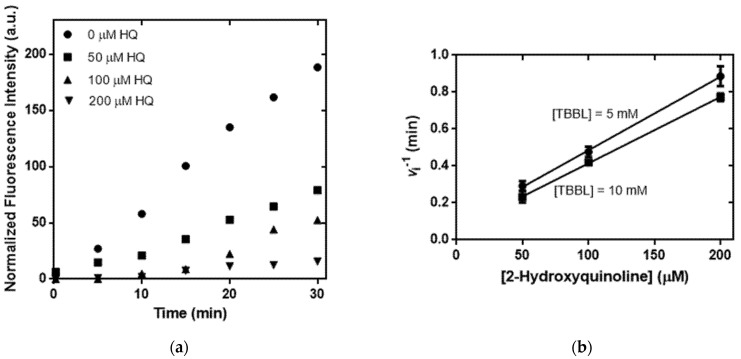
2-Hydroxyquinoline (HQ) inhibition of PON1 catalysis analyzed by the fluorescence assay based on **16b**. (**a**) Time-course kinetic analysis of HQ inhibition of PON1 (250.9 U L^−1^, 1.25% glycerol) catalysis was performed in the presence of TBBL (5 mM in ACN, 1%) in the Tris buffer. Each PON1 reaction contained 0, 50, 100 or 200 μM of HQ (in 1% ACN). (**b**) Dixon plot of the inhibitory kinetics of HQ on PON1 catalysis was derived from the *v*_i_ in (**a**) and Figure S5. The HQ inhibitor constant *K*_i_ was determined from the minus x-axis value at which the two extrapolated lines intersected, which, in this study, was 20.9 μM. Please see Section 3.4 for the experimental details. Each reaction was analyzed at least three times in order to acquire the averaged values and standard deviation (the error bar) of *v*_i_.

We further explicated the importance of PON1 lactonase activity in the fluorescence turn-on analysis based on **16b** by measuring the inhibition of PON1 catalysis in the presence of an inhibitor. Here, we have studied the inhibitory effect of 2-hydroxyquinoline (HQ) on PON1 catalysis using the **16b**-based assay. HQ is a well-known PON1 inhibitor, which inhibits PON1 catalysis vis a competitive mechanism [30]. We successfully observed that the inhibitory assay based on **16b** was able to detect a gradual decrease in *v*_i_ with increasing HQ concentrations (Figure 6a and Figure S5, SM). Kinetic analysis of the HQ inhibition of PON1 catalysis allowed us to acquire a Dixon plot (Figure 6b), which was employed to estimate the *K*_i_ value of 20.9 μM, a result over ten-fold higher than the *K*_i_ value of 0.9–1.42 μM determined by colorimetric methods [21]. The higher value of *K*_i_ obtained from the current study might reflect the approximate nature of the Dixon plot analysis. Nevertheless, the results in Figure 6 and Figure S5 unmistakably demonstrate that the **16b**-based assay indeed requires PON1 lactonase activity to liberate the 6-FAM fluorescence, and is well positioned to sensitively measure the variable PON1 lactonase activities in serum crucial to the diagnosis and treatment of human diseases.

## 3. Materials and Methods

All reagent-grade chemicals were purchased from commercial sources (Sigma-Aldrich, Acros, Alfa Aesar, and Mallinckrodt Baker) except where noted, and were further purified as necessary. ^1^H and ^13^C NMR spectra were recorded using either a Varian 200 or 400 MHz spectrometer (Varian, Inc., Palo Alto, CA, USA) at Kaohsiung Medical University, Taiwan (KMU). NMR samples were prepared in (CD_3_)_2_SO or CDCl_3_, and the chemical shifts of ^1^H and ^13^C signals were reported in parts per million based on the internal standard of each deuterated solvent. ESI high-resolution mass spectra were acquired using Solarix Fourier-transfer mass spectrometry (Bruker Taiwan Co., Ltd., Taiwan) at the Department of Chemistry, National Sun Yat-Sen University. The plasmid pET32a-PON1-G2E6 [46] containing the rePON1 gene was a generous gift of D. S. Tawfik (Weizmann Institute of Science, Israel). Synthesis of ***exo*-6** has been reported previously [38] (Scheme S1, SM). TBBL, the substrate required for the measurement of PON1 lactonase activity, was synthesized by following a reported method [30].

### 3.1. Synthesis of Azido-Rhodamine B (***9***), Separation and Regeneration of 5-FAM (***13a***) and 6-FAM (***13b***) for Synthesis of the Corresponding Geometric Isomer-Pure N-Hydroxysuccinimide Ester Derivatives (***14***), and Synthesis of the Rhodamine B-Carboxyfluorescein (FAM) Paired Fluorescent Chemical Probes ***16***

#### 3.1.1. Synthesis of Azido-Rhodamine B (**9**)

Compound **8** was one of the reactants used to synthesize **9** and was obtained from **7** [47]. The synthesis of **9** began with dissolution of rhodamine B (149.2 mg, 0.337 mmol, 1 equiv) in a solution containing acetonitrile (ACN; 4 mL) and chloroform (1 mL). BOP (benzotriazol-1-yloxytris(dimethylamino)phosphonium hexafluorophosphate) reagent (372.6 mg, 0.842 mmol, 2.5 equiv), **8** (101.9 mg, 1.011 mmol, 3 equiv) and *N*,*N*-diisopropylethylamine (DIPEA; 600 μL, 3.444 mol, 10.2 equiv) were added in sequence to the rhodamine B solution to initiate the reaction in the dark at rt while stirring. The overnight reaction mixture was rotovapped, redissolved in a limited volume of a methanol (MeOH)/dichloromethane (DCM) (1:40) solution, and loaded onto a silica column pre-equilibrated with the same solution. Compounds were eluted out of the column in the dark; fractions containing the product were pooled and evaporated under reduced pressure to yield **9** (100.5 mg, 0.207 mmol, 61.4%). ^1^H NMR (400 MHz, CDCl_3_) δ: 7.93–7.88 (m, 1H), 7.46–7.42 (m, 2H), 7.11–7.07 (m, 1H), 6.44–6.39 (m, 4H), 6.29–6.26 (m, 2H), 3.33 (quint, 8H), 3.17 (t, 2H), 3.06 (t, 2H), 1.43 (quint, 2H), 1.17 (t, 12H). ^13^C NMR (100.67 MHz, CDCl_3_) δ: 168.1, 153.3, 148.8, 132.4, 131.2, 128.7, 128.0, 123.8, 122.7, 108.1, 105.5, 97.7, 64.9, 49.3, 44.3, 37.5, 27.7, 12.5. HRMS (ESI) calculated for C_31_H_37_N_6_O_2_, [M + H]^+^ 525.29725 (calcd.), 525.29749 (found).

#### 3.1.2. Separation and Regeneration of 5-FAM (**13a**) and 6-FAM (**13b**) for the Synthesis of the Corresponding Geometric Isomer-Pure N-Hydroxysuccinimide (NHS) Ester Derivatives (**14**)

##### 3′,6′-bis(isobutyryloxy)-5(6)-carboxyfluorescein (**11**), Diisopropylammonium 3′,6′-bis(isobutyryloxy)-5-carboxyfluorescein (**12a**) and Diisopropylammonium 3′,6′-bis(isobutyryloxy)-6-carboxyfluorescein (**12b**)

A previously described method was applied to isobutyrylate 5(6)-carboxyfluorescein (**10**) to give **11**, which was separated to give **12a** and **12b** [47].

##### 5-Carboxyfluorescein (5-FAM, **13a**)

The reaction was performed by dissolving **12a** (228.3 mg, 0.441 mmol, 1 equiv) in 5 mL of trifluoroacetic acid (TFA) and refluxing in the dark for 4 h. The reaction mixture was allowed to cool to rt and TFA was subsequently removed under reduced pressure. The resulting solid phase was worked up three times by the following procedure: resuspending in diethyl ether (Et_2_O; 3–5 mL), transferring to a test tube, centrifuging, and decanting the supernatant. The final solid was dissolved in MeOH (3 mL) and evaporated under reduced pressure to obtain **13a** (159.2 mg, 0.422 mmol, 95 %). ^1^H NMR (400 MHz, C_2_D_6_OS) δ: 8.44 (s, 1H), 8.30 (d, 1H), 7.20 (d, 1H), 6.71 (s, 2H), 6.54 (s, 4H). ^13^C NMR (100.67 MHz, C_2_D_6_OS) δ: 168.7, 160.2, 153.1, 151.9, 136.2, 129.0, 126.3, 125.2, 123.3, 112.9, 109.5, 102.3. HRMS (ESI) calculated for C_2__1_H_1__2_O_7_, [M + H]^+^ 377.06558 (calcd.), 377.06560 (found).

##### 6-Carboxyfluorescein (6-FAM, **13b**)

Synthesis of **13b** was similarly carried out by dissolving **12b** (184 mg, 0.356 mmol, 1 equiv) in 5 mL of TFA and refluxing in the dark for 4 h. The reaction mixture was again allowed to cool to rt, followed by removing TFA under reduced pressure. The resulting solid phase was resuspended in an Et_2_O/ hexane solution (2:3; 3–5 mL), transferred to a test tube, and centrifuged; lastly, the supernatant was decanted. The workup procedure was performed two more times before dissolving the remaining solid in MeOH (3 mL). The resulting MeOH solution was evaporated under reduced pressure to obtain **13b** (108 mg, 0.286 mmol, 80 %). ^1^H NMR (400 MHz, C_2_D_6_OS) δ: 8.22 (d, 1H), 8.10 (d, 1H), 7.66 (s, 1H), 6.70 (s, 2H), 6.61 (d, 2H), 6.55 (d, 2H). ^13^C NMR (100.67 MHz, C_2_D_6_OS) δ: 167.9, 166.1, 159.7, 152.7, 151.9, 137.3, 130.9, 129.5, 129.2, 125.3, 124.5, 112.7, 109.0, 102.3. HRMS (ESI) calculated for C_2__1_H_1__2_O_7_, [M + H]^+^ 377.06558 (calcd.), 377.06562 (found).

##### 5-FAM NHS Ester (**14a**)

Compound **13a** (176 mg, 0.467 mmol, 1 equiv), NHS (134 mg, 1.164 mmol, 2.5 equiv) and *N*-(3-dimethylaminopropyl)-*N*′-ethylcarbodiimide hydrochloride (EDC; 134 mg, 0.699 mmol, 1.5 equiv) were sequentially dissolved in 2 mL of dimethylformamide (DMF) to start the reaction, which was stirred and allowed to proceed in the dark at rt under nitrogen atmosphere overnight. To this final reaction mixture was added 1 mL of DMF, 5.5 mL of ACN, and 10 mL of NaH_2_PO_4_ (0.05 M), in sequence. The resulting solution was extracted three times with the ethyl acetate (EA)/Et_2_O (1:2) solution (18 mL), three times with double deionized water (20 mL), and a saturated brine (20 mL). The final organic phase was dried over MgSO_4_, filtered, and evaporated under reduced pressure to provide **14a** (189 mg, 0.399 mmol, 85 %). ^1^H NMR (400 MHz, C_2_D_6_OS) δ: 10.19 (brs, 2H), 8.55 (s, 1H), 8.43 (d, 1H), 7.56 (d, 1H), 6.72–6.69 (m, 4H), 6.56–6.54 (m, 2H), 2.93 (s, 4H). ^13^C NMR (100.67 MHz, C_2_D_6_OS) δ: 206.5, 170.1, 167.1, 160.8, 159.8, 159.6, 158.1, 151.9, 151.82, 136.5, 129.5, 127.6, 126.7, 126.4, 125.7, 112.7, 108.5, 102.3, 83.8, 30.6, 25.6. HRMS (ESI) calculated for C_25_H_16_NO_9_, [M + H]^+^ 474.08196 (calcd.), 474.08166 (found).

##### 6-FAM NHS Ester (**14b**)

The synthesis of **14b** was achieved by the addition of **13b** (100 mg, 0.265 mmol, 1 equiv), NHS (76 mg, 0.66 mmol, 2.5 equiv) and EDC (76 mg, 0.396 mmol, 1.5 equiv) in sequence to 2 mL of DMF. The acquired solution was similarly reacted and worked up by following the procedures for the **14a** synthesis reported above to yield **14b** (97.4 mg, 0.205 mmol, 77.4 %). ^1^H NMR (400 MHz, C_2_D_6_OS) δ: 10.19 (brs, 2H), 8.40 (d, 1H), 8.25 (d, 1H), 7.93 (s, 1H), 6.71–6.67 (m, 4H), 6.59–6.55 (m, 2H), 2.87 (s, 4H). ^13^C NMR (100.67 MHz, C_2_D_6_OS) δ: 206.5, 170.0, 167.3, 160.9, 159.8, 152.8, 152.0, 151.9, 131.8, 131.6, 130.7, 129.4, 126.3, 125.5, 112.8, 108.7, 102.4, 84.0, 30.7, 25.5, 25.2. HRMS (ESI) calculated for C_25_H_16_NO_9_, [M + H]^+^ 474.08196 (calcd.), 474.08178 (found).

#### 3.1.3. Synthesis of the Rhodamine B-FAM Paired Fluorescent Chemical Probes (**16**)

##### The *exo*–BCN-5-FAM Conjugate (**15a**)

For the synthesis of **15a**, ***exo*-6** (31.9 mg, 0.097 mmol, 1 equiv), **14a** (55.2 mg, 0.117 mmol, 1.2 equiv), and DIPEA (75.7 mg, 102 μL, 0.583 mmol, 6 equiv) were sequentially dissolved in 2 mL of DMF to initiate the reaction, which was stirred and allowed to proceed in the dark at rt in a nitrogen atmosphere overnight. The resulting reaction mixture was evaporated under reduced pressure, redissolved in a limited volume of an acetic acid (AcOH)/MeOH/DCM (1:25:75) solution, and loaded onto a silica column pre-equilibrated with the same solution. Compounds were eluted from the column in the dark; fractions containing the product were pooled and evaporated under reduced pressure to give **15a** (53.4 mg, 0.078 mmol, 80.2%). ^1^H NMR (400 MHz, C_2_D_6_OS) δ: 8.45 (s, 1H), 8.23 (d, 1H), 7.37 (d, 1H), 6.68 (d, 2H), 6.56 (q, 4H), 3.85 (d, 2H), 3.61 (q, 2H), 3.28 (q, 2H), 2.95 (t, 2H), 2.81 (t, 2H), 2.29–2.02 (m, 7H), 1.33–1.21 (m, 3H), 0.67–0.60 (m, 3H). ^13^C NMR (100.67 MHz, C_2_D_6_OS) δ: 172.4, 168.3, 164.9, 162.5, 160.0, 156.5, 154.5, 152.0, 136.l, 134.6, 129.2, 126.9, 124.5, 123.5, 113.0, 109.2, 102.4, 99.0, 68.0, 54.9, 37.7, 37.1, 35.9, 32.9, 30.9, 23.4, 22.3, 21.4, 20.9. HRMS (ESI) calculated for C_36_H_33_N_2_O_8_S_2_, [M − H]^−^ 685.16838 (calcd.), 685.16824 (found).

##### The *exo*–BCN-6-FAM Conjugate (**15b**)

Similarly, ***exo*-6** (40.6 mg, 0.124 mmol, 1 equiv), **14b** (70.2 mg, 0.148 mmol, 1.2 equiv), and DIPEA (96.1 mg, 129.5 μL, 0.742 mmol, 6 equiv) were sequentially dissolved in 2 mL of DMF; the reaction proceeded and the products treated following the same procedures as were used for **15a** synthesis. The reaction described in this section afforded **15b** (52.2 mg, 0.761 mmol, 61.6%). ^1^H NMR (400 MHz, C_2_D_6_OS) δ: 8.17 (d, 1H), 8.08 (d, 1H), 7.67 (s, 1H), 6.70 (d, 2H), 6.58 (q, 4H), 3.83 (d, 2H), 3.49 (q, 2H), 3.22 (q, 2H), 2,74 (t, 2H), 2.29–2.03 (m, 7H), 1.29–1.22 (m, 3H), 0.67–0.58 (m, 3H). ^13^C NMR (100.67 MHz, C_2_D_6_OS) δ: 168.0, 164.6, 162.3, 159.8, 156.4, 152.5, 151.9, 140.4, 129.3, 128.5, 125.0, 122.3, 112.8, 109.2, 102.3, 98.9, 67.8, 55.8, 38.9, 37.5, 36.7, 35.8, 32.8, 30.8, 29.6, 23.4, 22.2, 20.8. HRMS (ESI) calculated for C_36_H_33_N_2_O_8_S_2_, [M − H]^−^ 685.16838 (calcd.), 685.16818 (found).

##### The Rhodamine B-5-FAM Paired Fluorescent Chemical Probe (**16a**)

Dioxane (2 mL) was used to dissolve **15a** (47.7 mg, 0.070 mmol, 1 equiv) and **9** (37 mg, 0.070 mmol, 1 equiv) and to give the reaction mixture, which was refluxed in the dark for 5 h. The resulting reaction mixture was evaporated under reduced pressure, redissolved in a limited volume of an AcOH/hexane/EA (1:10:100) solution, and loaded onto a silica column pre-equilibrated with the same solution to wash out impurities in the dark. Additional compounds were eluted out of the column by an AcOH/EA (1:100) solution; fractions containing the product were pooled and evaporated under reduced pressure to give **16a** (80.5 mg, 0.066 mmol, 95%). ^1^H NMR (400 MHz, C_2_D_6_OS) δ: 9.01 (t, 1H), 8.50 (s, 1H), 8.12 (d, 1H), 7.78–7.63 (m, 1H), 7.49–7.48 (m, 2H), 7.32–7.28 (m, 2H), 7.00–6.99 (m, 1H), 6.64–6.43 (m, 6H), 6.35–6.29 (m, 6H), 4.02 (t, 2H), 3.59 (t, 2H), 3.31–3.28 (m, 12H), 2.98–2.96 (m, 4H), 2.86–2.72 (m, 6H), 2.60–2.55 (m, 4H), 2.35–2.11 (m, 7H), 1.80–1.73 (m, 10H), 1.50 (quint, 2H), 1.21–1.11 (m, 3H), 1.07 (t, 12H), 0.76–0.67 (m, 3H). ^13^C NMR (100.67 MHz, C_2_D_6_OS) δ: 174.4, 168.5, 167.0, 165.4, 162.4, 156.4, 153.9, 153.4, 152.7, 148.4, 143.9, 139.2, 135.4, 132.8, 130.4, 129.5, 128.3, 124.9, 123.6, 122.3, 116.5, 109.8, 108.2, 104.8, 102.5, 97.2, 67.4, 67.0, 65.7, 64.1, 45.5, 43.7, 37.6, 37.2, 37.1, 35.8, 34.4, 30.8, 30.4, 28.5, 26.9, 26.1, 25.2, 23.8, 23.0, 22.1, 21.6, 12.4. HRMS (ESI) calculated for C_67_H_71_N_8_O_10_S_2_, [M − H]^−^ 1211.47291 (calcd.), 1211.47273 (found).

##### The Rhodamine B-6-FAM Paired Fluorescent Chemical Probe (**16b**)

The method of **16a** synthesis described above was analogously applied for the synthesis **16b**. Here a dioxane (2 mL) solution containing **15b** (40 mg, 0.058 mmol, 1 equiv) and **9** (30.6 mg, 0.058 mmol, 1 equiv) was reacted and worked out to yield **16b** (63.1 mg, 0.052 mmol, 89.4%). ^1^H NMR (400 MHz, C_2_D_6_OS) δ: 8.85 (t, 1H), 8.09 (q, 2H), 7.78–7.76 (m, 1H), 7.66 (s, 1H), 7.51–7.46 (m, 2H), 7.26 (t, 1H), 7.01–6.99 (m, 1H), 6.61 (d, 2H), 6.54 (s, 2H), 6.44 (d, 2H), 6.35–6.23 (m, 6H), 4.01 (t, 2H), 3.52–3.48 (m, 2H), 3.33–3.23 (m, 12H), 2.96 (t, 3H), 2.88 (t, 4H), 2.75 (t, 2H), 2.34–2.08 (m, 4H), 1.50 (quint, 2H), 1.27 (s, 3H), 1.06 (t, 12H), 0.78–0.66 (m, 4H). ^13^C NMR (100.67 MHz, C_2_D_6_OS) δ: 172.9, 168.1, 166.9, 164.8, 162.3, 156.3, 153.7, 153.4, 152.7, 148.4, 143.9, 138.0, 132.8, 132.7, 130.4, 129.6, 128.7, 128.3, 126.7, 124.2, 123.6, 122.3, 116.1, 109.9, 108.1, 104.8, 102.4, 97.2,67.4, 64,1, 48.6, 45.4, 43.7, 37.5, 37.2, 36.7, 35.7, 29.0, 28.4, 26.8, 26.0, 25.2, 23.7, 22.3, 22.1, 21.6, 12.4. HRMS (ESI) calculated for C_67_H_69_N_8_O_10_S_2_, [M − H]^−^ 1209.45836 (calcd.), 1209.45827 (found).

### 3.2. Spectroscopic Measurements

The UV-Vis absorption spectra and kinetic studies were recorded using an Amersham Biosciences Ultrospec 2100 pro spectrophotometer (KMU), and fluorescence spectra and kinetic studies were obtained with a PerkinElmer LS-55 fluorescence spectrometer (KMU) with an excitation wavelength of 325 nm for 6-FAM detection in a 1 cm standard quartz cuvette.

### 3.3. Specificity and Mechanism of the Reactions of the Fluorescent Chemical Probe ***16b*** with Thiols

The preference of **16b** to react with certain compounds was characterized by the obtained pseudo-first order rate constant (*k*_1_) values. A typical experiment for *k*_1_ determination is briefly described below. A quartz cuvette containing 1388 μL of a Tris buffer (50 mM of Tris, 1 mM of Ca^2+^, pH 8.0) was mounted onto the temperature controller of the PerkinElmer LS-55 fluorescence spectrometer first. The measurement was initiated after the sequential transfer of 150 μL of a 50 mM solution of one of the reactants (DL-dithiothreitol (DTT), L-glutamate, glycine, L-cysteine, GSH, L-serine, L-lysine, L-methionine, 2-mercaptoethanol, 2-AET, or nBuSH) in the Tris buffer and 12 μL of **16b** (50 μM in DMF) to the Tris solution in the cuvette to give reactant = 5 mM and **16b** = 0.4 μM, respectively, in the reaction. The progress of the reaction at 25 °C was detected by the liberation of the 6-FAM fluorescence (λ_max_ = 525 nm) until signal saturation occurred, typically after 15–90 min. The data of normalized fluorescence intensity at 525 nm were acquired by subtracting the background fluorescence intensity at 525 nm of **16b** from the original measurement of fluorescence intensity at 525 nm. The normalized data were fitted to a single-exponential equation for first-order kinetics F(*t*) = F_0_ + F*_max_*(1 − e^−*k*1*t*^) (F(*t*), normalized 6-FAM fluorescence at a specific time point *t*) to give *k*_1_ (GraphPad, La Jolla, CA, USA). Similar kinetic analysis was exploited to determine *k*_2,_ in which the concentration of 2-AET in the reaction was 1 mM, 2.5 mM, 5 mM, 10 mM, 12.5 mM or 15 mM. A second-order rate constant (*k*_2_) value was acquired from the slope of a linear regression curve based on the plot of *k*_1_ vs. 2-AET. The pH effect on the reaction of **16b** with 2-AET was determined by the same first-order kinetics in the presence of different pH buffers including MES (pH 5.5–6.5), PIPES (pH 6.5–7.4), EPPS (pH 7.4–8.6) and CHES (pH 8.6–9.5). The pH titration data enabled calculation of the p*K*_a1_ value for 2-AET according to the equation *k*_1_ = *k*_1,max_ [1 + 10^(p*K*a1−pH)^] (GraphPad, USA). The similar *k*_1_ determination of the L-cysteine**-16b** reactions in the presence of 1 mM of metal ions (K^+^, Li^+^, Na^+^, Cd^2+^, Co(II), Cu(II), Mg^2+^, Mn(II), Ni(II), Zn^2+^, Fe(III)) and the background of 1 mM Ca^2+^ unveiled the effects of metal ions on the reaction. The potential of **16b** to react discriminately against GSH was likewise evaluated by the first-order kinetics of the reactions between **16b** and 5 mM of the mono-ethyl (**17**) and the dimethyl (**18**) ester derivatives of GSH [38]. Kinetic experiments were performed in quadruplicate in order to determine *k*_1_ for a specific reaction; the provided *k*_1_ was the mean ± SD of the four experiments.

### 3.4. Development of Fluorescence Turn-On Assay Based on ***16b*** and Able to Measure PON1 Lactonase Activity in Serum and in the Presence of a PON1 Inhibitor

The plasmid pET32a-PON1-G2E6 was transformed into BL21(DE3) cells in order to overexpress rePON1, which was shown to have a catalytic activity similar to human PON1 [46], and was subsequently purified according to an established method [46]. The purified rePON1 protein concentration was determined by the Bradford assay (Bio-Rad), and rePON1 lactonase activity was calibrated using Ellman’s colorimetric method [48]. PON1 lactonase activity is expressed as U L^−1^ in which one unit (1 U) is defined as the amount of PON1 able to hydrolyze 1 μmol of TBBL per min. Analysis of PON1 activity for different PON1 samples in the presence of **16b** was performed qualitatively in vials and quantitatively in the fluorescence spectrometer. The vial assay included four different solutions in which each vial contained a different combination of 2 μM of **16b** (dissolved in dimethyl sulfoxide (DMSO), 0.5%), 5 mM of TBBL (dissolved in ACN, 0.2%), and 7.79 U L^−1^ of rePON1 (50% glycerol) in the Tris buffer. The solutions were prepared in the dark in four different vials and reacted at rt in the dark for 15 min. The liberated fluorescence was visualized and photographed, while the samples were illuminated with 365 nm light from a hand-held lamp.

PON1 lactonase activity was quantitatively determined by utilizing the **16b**-based assay in solutions containing **16b** (0.8 μM in DMSO, 1.6%), rePON1 (8.6–232.1 U L^−1^, 0.25–1.25% glycerol), and TBBL (10 mM in ACN, 1%) in the Tris buffer in a quartz cuvette previously mounted onto the temperature controller of the PerkinElmer LS-55 fluorescence spectrometer. PON1 catalysis was carried out at 25 °C for 30 min; reaction progression was followed by monitoring the fluorescence intensity at 525 nm as a function of reaction time. The normalized fluorescence intensity data at 525 nm were acquired by subtracting a background fluorescence of **16b** at the same wavelength from the original fluorescence intensity reading. Initial velocities (*v*_i_) of PON1 catalysis were calculated from the fluorescence changes in windows of the steady state reactions. Human PON1 lactonase activity was measured after a trained phlebotomist at KMU Hospital obtained 20 mL of whole blood from three healthy male volunteers; the blood samples were centrifuged, the serum was separated from the plasma and diluted 100-fold, and the diluted serum fraction was used as a substitute for rePON1 in separate **16b**-based reactions. It is noted that, before the addition of TBBL (10 mM in ACN, 1%), GSH in diluted serum was consumed in the presence of **16b** (0.8 μM in DMSO, 1.6%) in the Tris buffer at 25 °C for 30 min. Finally, effects of the inhibitor HQ on PON1 catalysis were studied viz **16b**-based analysis, in which each reaction contained **16b** (0.8 μM in DMSO, 1.6%), rePON1 (250.9 U L^−1^, 1.25% glycerol), TBBL (5 or 10 mM, 1% or 1.5% ACN, respectively), and HQ (50, 100, or 200 μM in ACN, 1% or 1.5%) in the Tris buffer. Each specific PON1 catalysis was performed three times with the reported *v*_i_ as a mean ± SD of the experiments.

## 4. Conclusions

In summary, the current study has successfully provided critical information essential to: (1) the effective organic synthesis of the chemical probes **16** enabled by the modulated bioorthogonal chemistry; (2) the demonstration of the importance of contact fluorescence quenching and disulfide cleavage on the fluorogenic properties of **16**; (3) the development of the first fluorescence turn-on assay for sensitive and accurate quantification of PON1 lactonase activity in samples of human serum. The assay exploited the fluorogenic property of the novel chemical probe **16b** in the presence of PON1 and TBBL to release the 6-FAM fluorescence, and delivered a linear detection range of 10.8–232.1 U L^−1^ and an LOD of 10.8 U L^−1^ in the measurement of PON1 lactonase activity. The specificity and sensitivity of the **16b**-based assay thus provides an excellent method to accurately and dynamically quantify PON1 lactonase activity in samples with broad applications in clinical settings and basic research.

The first major contribution of the study is highlighted by the synthetic method used for the chemical probes **16**. Here, we developed a facile approach to separate 5-FAM and 6-FAM, which proved to be critical to improving the sensitivity of chemical probes in the study (Scheme 3). In addition, we have shown that the two-reaction method for the synthesis of **16** (Scheme 4) was more effective than the previously reported one-pot format [38], and provided higher yields when covalently linking two different chromophores to ***exo*-6** in a programmed manner. The higher yield provided by the two-reaction approach in the synthesis of **16** is attributed to the outstanding reactivity of the SPAAC reaction to rapidly generate a triazole product, which, however, might be bulky enough to prevent subsequent amidation reactions with electrophiles. The synthesis of a chemical probe with high yields may require amidation reactions of electrophiles with ***exo*-6** to be performed first, followed by the SPAAC reaction. We are collaborating with colleagues to determine the X-ray structures of **16**. The molecular structures of **16** will provide crucial information about not only the extent of molecular crowdedness in **16,** but also the importance of contact quenching effects on the fluorogenic properties of **16,** which will be discussed in the next paragraph.

The second significant aspect of this study is that it provides evidence associating contact effects with the quenching of the FAM fluorescence in the chemical probes **16** (Figure 1a). The UV-Vis spectra support the assertion that the rhodamine B and FAM moieties in **16** were in van der Waals contact, in order to enable efficient FAM fluorescence quenching due to the distance between the fluorophores likely being on the sub-nanometer length scale. Moreover, the distance between rhodamine B and 6-FAM in **16b** is probably shorter than that between rhodamine B and 5-FAM in **16a**. The subtle distance difference between rhodamine B and FAM in **16** was enough to enhance contact quenching in **16b** more prominently than in **16a,** and to establish **16b** as the more sensitive chemical probe (Figure 1b and Figure S1A, SM). Again, elucidating the X-ray structures of **16a** and **16b** will help us to better understand the dominant role of contact quenching in **16,** and to decipher the peculiar but unique reactivity of **16b** with nBuSH.

We were concerned that the rather low reactivity of **16b** with nBuSH would somehow affect the sensitivity of the fluorescence turn-on assay for the quantification of PON1 lactonase activity (Figure 2 and Figure S2, SM). The differential reactivity of **16b** with various thiolates, including nBuSH, could not be solely explained by steric hindrance, electrostatic interactions, or a hydrophobic effect, and was likely a combination of multiple factors simultaneously. Nevertheless, **16b** preferentially reacted with small, charged, and hydrophilic thiolates. Therefore, based on the understanding of the fluorogenic reaction mechanism for **16b** (Figure 3 and Figure S3, SM), we are synthesizing better lactonase substrates to substitute for TBBL in PON1 catalysis, in order to further improve the sensitivity and accuracy of the PON1 lactonase activity assay.

Finally, this study has clearly demonstrated the development and use of the first fluorescence turn-on assay to accurately quantify PON1 lactonase activity in samples including sera (Figure 4, Figure 5 and Figure S4, SM). The fluorogenic property of the assay exclusively depends on the PON1 lactonase activity releasing nBuSH, which was subsequently able to nucleophilically attack the disulfide bond in **16b** (Figure 6 and Figure S5, SM). As stated above, we are undertaking on-going studies aimed at developing fluorescence assays that will measure PON1 lactonase activity more sensitively and accurately. The critical information provided by current and future studies will pave the way for attaining high-throughput diagnostic tools capable of efficiently and effectively detecting aberrant PON1 lactonase activity in serum and other biological samples, so as to facilitate the diagnosis and treatment of human diseases.

## Data Availability

Not applicable.

## Supplementary Materials

The following are available online at https://www.mdpi.com/article/10.3390/molecules27082435/s1. Scheme S1: Synthesis of the key bicyclononyne derivatives (**6**). Figure S1: The fluorogenic property of the chemical probe **16a** when reacting with a thiol and the presence of residual FRET effects in **16**. Figure S2: Representative pseudo-first order reactions of **16b** (0.4 μM) with 5 mM of (A) 2-AET, (B) DTT, (C) L-cysteine, (D) GSH, (E) nBuSH, (F) 2-ThioEtOH, or (G) five non-thiol amino acids (L-methionine, L-lysine, L-serine, L-glutamate, and glycine) in the Tris buffer (50 mM Tris, 1 mM Ca^2+^, pH 8.0). Figure S3: Influence of metal ions and steric constraints of **16b** on the thiol-dependent fluorogenic reaction of **16b**. Figure S4: Presence of PON1 and TBBL is required for the fluorogenic property of **16b**. Figure S5: 2-Hydroxyquinoline (HQ) inhibition on PON1 catalysis analyzed by the fluorescence assay based on **16b**. NMR and HRMS spectra for compounds (**9, 13, 14, 15** and **16**). References [38,39] are cited in the supplementary materials.

## Abbreviations

ACN: acetonitrile; 2-AET, 2-aminoethanethiol; BChE, butyrylcholinesterase; DMF, dimethylformamide; DMSO, dimethyl sulfoxide; DTT, DL-dithiothreitol; 5-FAM, 5-carboxyfluorescein; 6-FAM, 6-carboxyfluorescein; FRET, Förster resonance energy transfer; GSH, glutathione; HDL, high-density lipoproteins; HQ, 2-hydroxyquinoline; LDL, low-density lipoproteins; LOD, limit of detection; nBuSH, 1-butanethiol; PON, paraoxonase; SPAAC, strained-promoted azide-alkyne cycloaddition; TBBL, 5-(thiobutyl) butyrolactone.

## References

[1] Moya C., Máñez S. (2018). Paraoxonases: Metabolic role and pharmacological projection. Naunyn-Schmiedebergs Arch. Pharmakol..

[2] Szczeklik K., Mach T., Cibor D., Owczarek D., Sapa J., Papież M., Pytko-Polończyk J., Krzyściak W. (2018). Correlation of Paraoxonase-1 with the Severity of Crohn’s Disease. Molecules.

[3] Camps J., Iftimie S., García-Heredia A., Castro A., Joven J. (2017). Paraoxonases and infectious diseases. Clin. Biochem..

[4] Baker R.G., Hayden M.S., Ghosh S. (2011). NF-κB, Inflammation, and Metabolic Disease. Cell Metab..

[5] Meneses M.J., Silvestre R., Sousa-Lima I., Macedo M.P. (2019). Paraoxonase-1 as a Regulator of Glucose and Lipid Homeostasis: Impact on the Onset and Progression of Metabolic Disorders. Int. J. Mol. Sci..

[6] Bigagli E., Lodovici M. (2019). Circulating Oxidative Stress Biomarkers in Clinical Studies on Type 2 Diabetes and Its Complications. Oxidative Med. Cell. Longev..

[7] Ferrín G., Rodríguez-Perálvarez M., Aguilar-Melero P., Ranchal I., Llamoza C., Linares C.I., González-Rubio S., Muntané J., Briceño J., López-Cillero P. (2015). Plasma Protein Biomarkers of Hepatocellular Carcinoma in HCV-Infected Alcoholic Patients with Cirrhosis. PLoS ONE.

[8] Jin Y., Yang Y., Su Y., Ye X., Liu W., Yang Q., Wang J., Fu X., Gong Y., Sun H. (2019). Identification a novel clinical biomarker in early diagnosis of human non-small cell lung cancer. Glycoconj. J..

[9] Shah A.K., Hartel G., Brown I., Winterford C., Na R., Cao K.-A.L., Spicer B.A., Dunstone M.A., Phillips W.A., Lord R.V. (2018). Evaluation of Serum Glycoprotein Biomarker Candidates for Detection of Esophageal Adenocarcinoma and Surveillance of Barrett’s Esophagus. Mol. Cell. Proteom..

[10] Yu Z., Ou Q., Chen F., Bi J., Li W., Ma J., Wang R., Huang X. (2018). Evaluation of the prognostic value of paraoxonase 1 in the recurrence and metastasis of hepatocellular carcinoma and establishment of a liver-specific predictive model of survival. J. Transl. Med..

[11] Aviram M., Rosenblat M., Bisgaier C.L., Newton R.S., Primo-Parmo S.L., La Du B.N. (1998). Paraoxonase inhibits high-density lipoprotein oxidation and preserves its functions. A possible peroxidative role for paraoxonase. J. Clin. Investig..

[12] Dias C.G., Batuca J.R., Marinho A.T., Caixas U., Monteiro E.C., Antunes A.M.M., Pereira S.A. (2013). Quantification of the arylesterase activity of paraoxonase-1 in human blood. Anal. Methods.

[13] Furlong C.E., Marsillach J., Jarvik G.P., Costa L.G. (2016). Paraoxonases-1, -2 and -3: What are their functions?. Chem. Interact..

[14] Davies H.G., Richter R.J., Keifer M., Broomfield C.A., Sowalla J., Furlong C.E. (1996). The effect of the human serum paraoxonase polymorphism is reversed with diazoxon, soman and sarin. Nat. Genet..

[15] Sorenson R.C., Bisgaier C.L., Aviram M., Hsu C., Billecke S., La Du B.N. (1999). Human Serum Paraoxonase/Arylesterase’s Retained Hydrophobic *N*-Terminal Leader Sequence Associates with HDLs by Binding Phospholipids. Arter. Thromb. Vasc. Biol..

[16] Harel M., Aharoni A., Gaidukov L., Brumshtein B., Khersonsky O., Meged R., Dvir H., Ravelli R., McCarthy A., Toker L. (2004). Structure and evolution of the serum paraoxonase family of detoxifying and anti-atherosclerotic enzymes. Nat. Struct. Mol. Biol..

[17] Mackness M., Mackness B. (2015). Human paraoxonase-1 (PON1): Gene structure and expression, promiscuous activities and multiple physiological roles. Gene.

[18] Reddy S.T., Wadleigh D.J., Grijalva V., Ng C., Hama S., Gangopadhyay A., Shih D.M., Lusis A.J., Navab M., Fogelman A.M. (2001). Human Paraoxonase-3 Is an HDL-Associated Enzyme With Biological Activity Similar to Paraoxonase-1 Protein but Is Not Regulated by Oxidized Lipids. Arter. Thromb. Vasc. Biol..

[19] Kuo C.L., La Du B.N. (1998). Calcium binding by human and rabbit serum paraoxonases. Structural stability and enzymatic activity. Drug Metab. Dispos..

[20] Draganov D.I., Teiber J.F., Speelman A., Osawa Y., Sunahara R., La Du B.N. (2005). Human paraoxonases (PON1, PON2, and PON3) are lactonases with overlapping and distinct substrate specificities. J. Lipid Res..

[21] Khersonsky O., Tawfik D.S. (2005). Structure−Reactivity Studies of Serum Paraoxonase PON1 Suggest that Its Native Activity Is Lactonase. Biochemistry.

[22] Jakubowski H. (2000). Calcium-dependent Human Serum Homocysteine Thiolactone Hydrolase. A protective mechanism against protein N-homocysteinylation. J. Biol. Chem..

[23] Mackness M.I., Abbott C., Arrol S., Durrington P.N. (1993). The role of high-density lipoprotein and lipid-soluble antioxidant vitamins in inhibiting low-density lipoprotein oxidation. Biochem. J..

[24] Mackness M., Arrol S., Abbott C., Durrington P. (1993). Protection of low-density lipoprotein against oxidative modification by high-density lipoprotein associated paraoxonase. Atherosclerosis.

[25] Gugliucci A., Caccavello R., Kotani K., Sakane N., Kimura S. (2013). Enzymatic assessment of paraoxonase 1 activity on HDL subclasses: A practical zymogram method to assess HDL function. Clin. Chim. Acta.

[26] Teiber J.F., Draganov D.I., Du B.N. (2003). Lactonase and lactonizing activities of human serum paraoxonase (PON1) and rabbit serum PON3. Biochem. Pharmacol..

[27] Togawa T., Mukai Y., Ohata K., Suzuki T., Tanabe S. (2005). Measurement of homocysteine thiolactone hydrolase activity using high-performance liquid chromatography with fluorescence detection and polymorphisms of paraoxonase in normal human serum. J. Chromatogr. B.

[28] Yeung D.T., Smith J.R., Sweeney R.E., Lenz D.E., Cerasoli D.M. (2007). Direct detection of stereospecific soman hydrolysis by wild-type human serum paraoxonase. FEBS J..

[29] Billecke S., Draganov D., Counsell R., Stetson P., Watson C., Hsu C., La Du B.N. (2000). Human serum paraoxonase (PON1) isozymes Q and R hydrolyze lactones and cyclic carbonate esters. Drug Metab. Dispos..

[30] Khersonsky O., Tawfik D.S. (2006). Chromogenic and Fluorogenic Assays for the Lactonase Activity of Serum Paraoxonases. ChemBioChem.

[31] Richter R.J., Jarvik G.P., Furlong C.E. (2009). Paraoxonase 1 (PON1) status and substrate hydrolysis. Toxicol. Appl. Pharmacol..

[32] Roodveldt C., Tawfik D.S. (2005). Shared Promiscuous Activities and Evolutionary Features in Various Members of the Amidohydrolase Superfamily. Biochemistry.

[33] Gaidukov L., Tawfik D.S. (2007). The development of human sera tests for HDL-bound serum PON1 and its lipolactonase activity. J. Lipid Res..

[34] Mu X., Yu N., Wang C., Zou X., Abulimite Z., Xia Z. (2011). Evaluation of a new substrate for measurement of serum PON arylesterase activity. Talanta.

[35] Ahmad S., Carter J.J., Scott J.E. (2010). A homogeneous cell-based assay for measurement of endogenous paraoxonase 1 activity. Anal. Biochem..

[36] Garai-Ibabe G., Möller M., Pavlov V. (2012). Ultrasensitive Assay for Detection of Serum Paraoxonase by Modulating the Growth of Fluorescent Semiconductor Nanoparticles. Anal. Chem..

[37] Soukharev S., Hammond D.J. (2004). A fluorogenic substrate for detection of organophosphatase activity. Anal. Biochem..

[38] Gong M.-M., Dai C.-Y., Severance S., Hwang C.-C., Fang B.-K., Lin H.-B., Huang C.-H., Ong C.-W., Wang J.-J., Lee P.-L. (2020). A Bioorthogonally Synthesized and Disulfide-Containing Fluorescence Turn-On Chemical Probe for Measurements of Butyrylcholinesterase Activity and Inhibition in the Presence of Physiological Glutathione. Catalysts.

[39] Dommerholt J., Schmidt S., Temming R., Hendriks L.J.A., Rutjes F.P.J.T., van Hest J.C.M., Lefeber D.J., Friedl P., van Delft F.L. (2010). Readily Accessible Bicyclononynes for Bioorthogonal Labeling and Three-Dimensional Imaging of Living Cells. Angew. Chem. Int. Ed..

[40] Johansson M.K., Fidder H., Dick D., Cook R.M. (2002). Intramolecular Dimers: A New Strategy to Fluorescence Quenching in Dual-Labeled Oligonucleotide Probes. J. Am. Chem. Soc..

[41] Marras S.A.E., Kramer F.R., Tyagi S. (2002). Efficiencies of fluorescence resonance energy transfer and contact-mediated quenching in oligonucleotide probes. Nucleic Acids Res..

[42] Takakusa H., Kikuchi K., Urano Y., Higuchi A.T., Nagano T. (2001). Intramolecular Fluorescence Resonance Energy Transfer System with Coumarin Donor Included in β-Cyclodextrin. Anal. Chem..

[43] Su Y.-C., Chen H.-Y., Ko N.C., Hwang C.-C., Wu M.H., Wang L.F., Wang Y.-M., Chang S.-N., Wang E.-C., Wang T.-P. (2014). Effective and site-specific phosphoramidation reaction for universally labeling nucleic acids. Anal. Biochem..

[44] Serjeant E.P., Dempsey B. (1979). Ionisation Constants of Organic Acids in Aqueous Solution.

[45] Jones D.P., Carlson J.L., Samiec P.S., Sternberg P., Mody V.C., Reed R.L., Brown L.A.S. (1998). Glutathione measurement in human plasma: Evaluation of sample collection, storage and derivatization conditions for analysis of dansyl derivatives by HPLC. Clin. Chim. Acta.

[46] Aharoni A., Gaidukov L., Yagur S., Toker L., Silman I., Tawfik D.S. (2004). Directed evolution of mammalian paraoxonases PON1 and PON3 for bacterial expression and catalytic specialization. Proc. Natl. Acad. Sci. USA.

[47] Horatscheck A., Wagner S., Ortwein J., Kim B.G., Lisurek M., Beligny S., Schütz A., Rademann J. (2012). Benzoylphosphonate-Based Photoactive Phosphopeptide Mimetics for Modulation of Protein Tyrosine Phosphatases and Highly Specific Labeling of SH2 Domains. Angew. Chem. Int. Ed..

[48] Ellman G.L., Courtney K.D., Andres V., Featherstone R.M. (1961). A new and rapid colorimetric determination of acetylcholinesterase activity. Biochem. Pharmacol..

