# Supplementary material for: Sensitive Assay for the Lactonase Activity of Serum Paraoxonase 1 (PON1) by Harnessing the Fluorescence Turn-On Characteristics of Bioorthogonally Synthesized and Geometrically Controlled Chemical Probes
Source: Molecules. 2022 Apr 9;27(8):2435. doi: 10.3390/molecules27082435 (PMC9027646; doi:10.3390/molecules27082435)
Supplement: Supplementary file 1 [file molecules-27-02435-s001.zip › molecules-1605057-supplementary.pdf]

Supplementary Materials

Sensitive assay for the lactonase activity of serum paraoxonase 1 (PON1) by harnessing the fluorescence turn-on characteristics of bioorthogonally synthesized and geometrically controlled chemical probes

Bo-Kai Fang [1,†], Chia-Yen Dai [2,3,†], Scott Severance [4], Chi-Ching Hwang [5], Chien-Hui Huang [1], Sin-Yu Hou [1], Bao-Lin Yeh [1], Ming-Mao Gong [1], Yun-Hao Chou[5], Jeh-Jeng Wang [1] and Tzu-Pin Wang [1,6,*]

* Corresponding author: tzupinw@kmu.edu.tw

* Contact address: Department of Medicinal and Applied Chemistry and Kaohsiung Medical University Hospital, Kaohsiung Medical University, Kaohsiung, 80708, Taiwan.

[†]Auhors contributed equally to this study.

Contents

1. Scheme S1…...…………………………..………………………..………………………S3

2. Figures S1-S5.. S4-S9

3. References.. S10

4. ^{1}H and ^{13}C NMR, and HRMS Spectra of Compounds.................................. S11-S37

Scheme

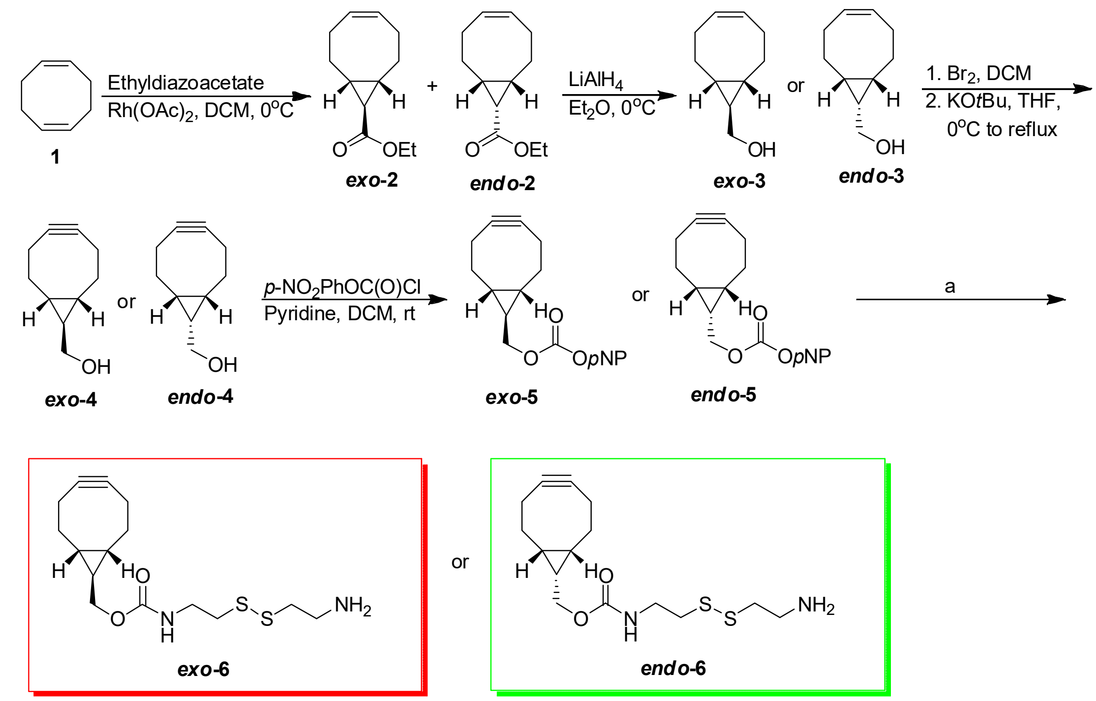

Scheme S1. Synthesis of the key bicyclononyne derivatives (**6**). Syntheses of **2** - **5** were reported previously [1, 2]. a: cystamine, Et_3N, MeOH/DCM, 50˚C.

Figures.

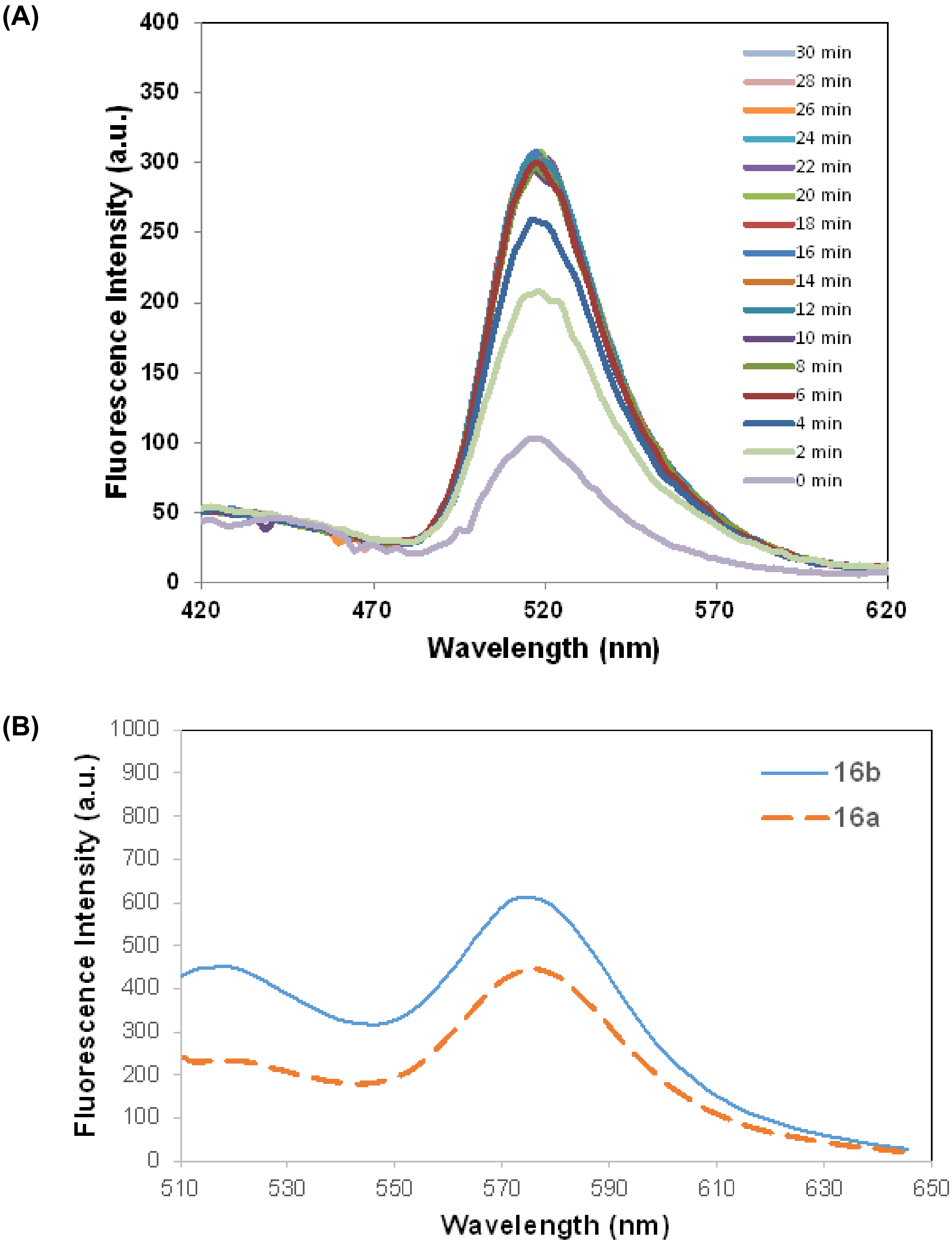

Figure S1. The fluorogenic property of the chemical probe **16a** when reacting with a thiol and the presence of residual FRET effects in **16**. (A) The time-dependent increase of the 5-FAM fluorescence in a reaction of **16a** (1 µM) with L-cysteine (50 mM) in phosphate buffer (PB; 10% DMF, pH 7.4). (B) The fluorescence spectra of **16** (4 µM each) in glacial acetic acid containing 0.8% of DMF. The excitation wavelength for **16a** was 494 nm and the excitation wavelength for **16b** was 491 nm.

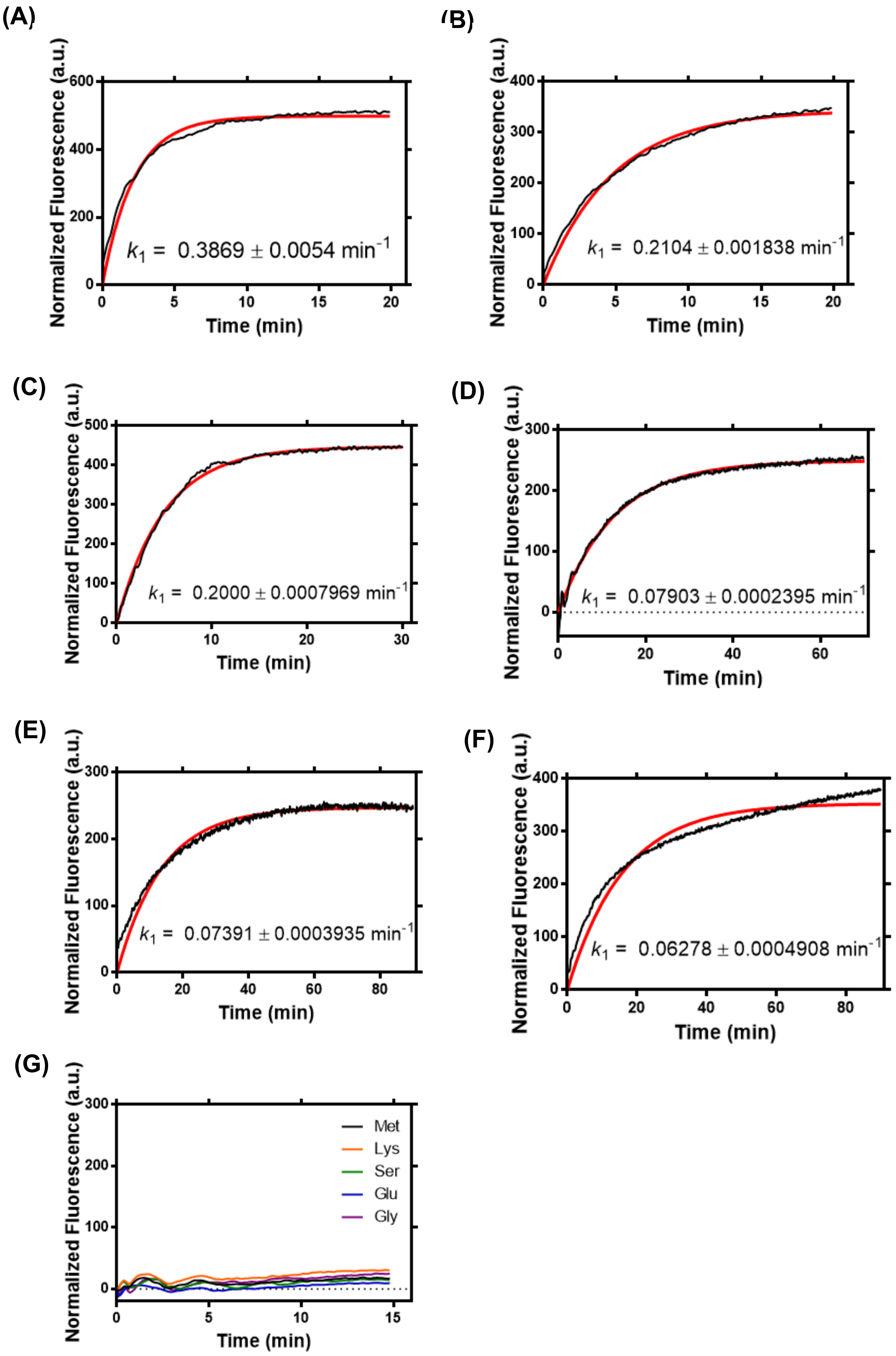
(A)
Normalized Fluorescence (a.u.)
Time (min)
k_1 = 0.3869 ± 0.0054 min^{-1}
(B)
Normalized Fluorescence (a.u.)
Time (min)
k_1 = 0.2104 ± 0.001838 min^{-1}
(C)
Normalized Fluorescence (a.u.)
Time (min)
k_1 = 0.2000 ± 0.0007969 min^{-1}
(D)
Normalized Fluorescence (a.u.)
Time (min)
k_1 = 0.07903 ± 0.0002395 min^{-1}
(E)
Normalized Fluorescence (a.u.)
Time (min)
k_1 = 0.07391 ± 0.0003935 min^{-1}
(F)
Normalized Fluorescence (a.u.)
Time (min)
k_1 = 0.06278 ± 0.0004908 min^{-1}
(G)
Normalized Fluorescence (a.u.)
Time (min)
Met
Lys
Ser
Glu
Gly

Figure S2. Representative pseudo-first-order reactions of **16b** (0.4 μM) with 5 mM of (A) 2-AET, (B) DTT, (C) L-cysteine, (D) GSH, (E) nBuSH, (F) 2-ThioEtOH, or (G) five non-thiol amino acids (L-methionine, L-lysine, L-serine, L-glutamate, and glycine) in the Tris buffer (50 mM Tris, 1 mM Ca^{2+}, pH 8.0). Progress of each reaction was monitored by measuring fluorescence emission at 525 nm at specific time intervals. Data of normalized 6-FAM fluorescence intensity (a.u.) *vs* time were fitted to a single-exponential equation for first-order kinetics $F(t) = F_0 + F_{max}(1 - e^{-k_1 t})$ [$F(t)$, 6-FAM fluorescence at a specific time point t] to provide the values of first-order rate constant k_1 (GraphPad, La Jolla, CA, USA) illustrated in the graphs. In Panels S3A-S3F, the black lines indicate original fluorescence changes in the reaction time courses, and the red curves show the results calculated by the single-exponential equation. The normalized fluorescence intensity data at 525 nm were acquired by subtracting a background fluorescence of **16b** from the original fluorescence intensity measurements. 2-AET, 2-aminoethanethiol; nBuSH, 1-butanethiol; 2-ThioEtOH, 2-mercaptoethanol.

(A)

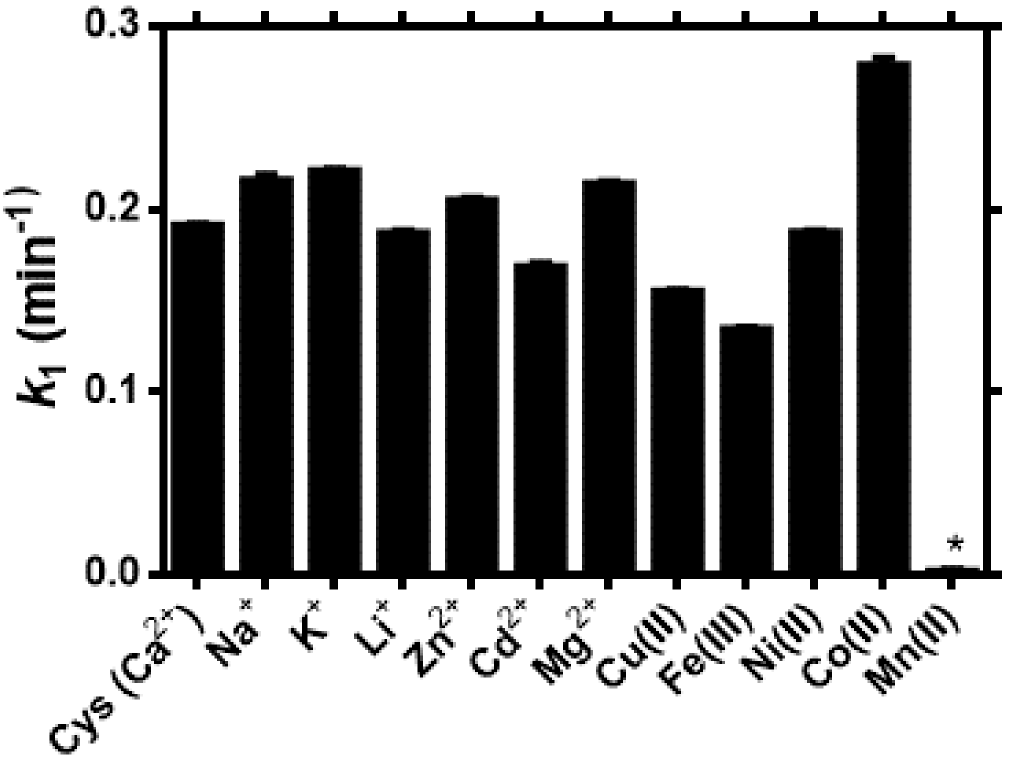

(B)

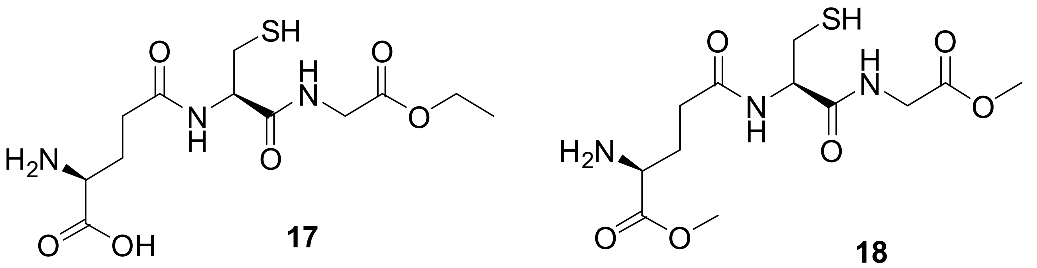

(C)

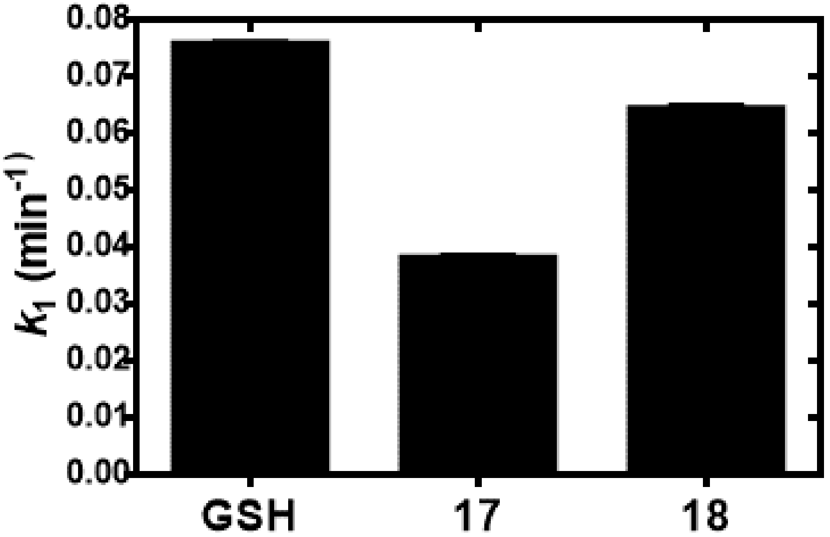

Figure S3. Influence of metal ions and steric constraints of **16b** on the thiol-dependent fluorogenic reaction of **16b**. (A) Effects of metal ion (1 mM) on the pseudo-first-order reactions of **16b** (0.4 μM) with 5 mM L-cysteine in the Tris buffer. The k_1 values were determined by a method analogous to what was described in Figure S2. The symbol * indicates that **16b** had no detectable 6-FAM fluorescence change in the presence of Mn(II) with which the 6-FAM fluorescence change was so insignificant that the values of averaged k_1 and standard deviation could not be determined. (B) Structures of two GSH derivatives **17** and **18** [2]. (C) Kinetic analysis of the pseudo-first-order reactions of **16b** (0.4 μM) with 5 mM of one of the structure-modified GSH derivatives **17** and **18** in the Tris buffer. The k_1 values were again determined by a method similar to that described in Figure S2. In addition, each reaction was analyzed at least three times in order to acquire the averaged k_1 values and standard deviation (the error bar).

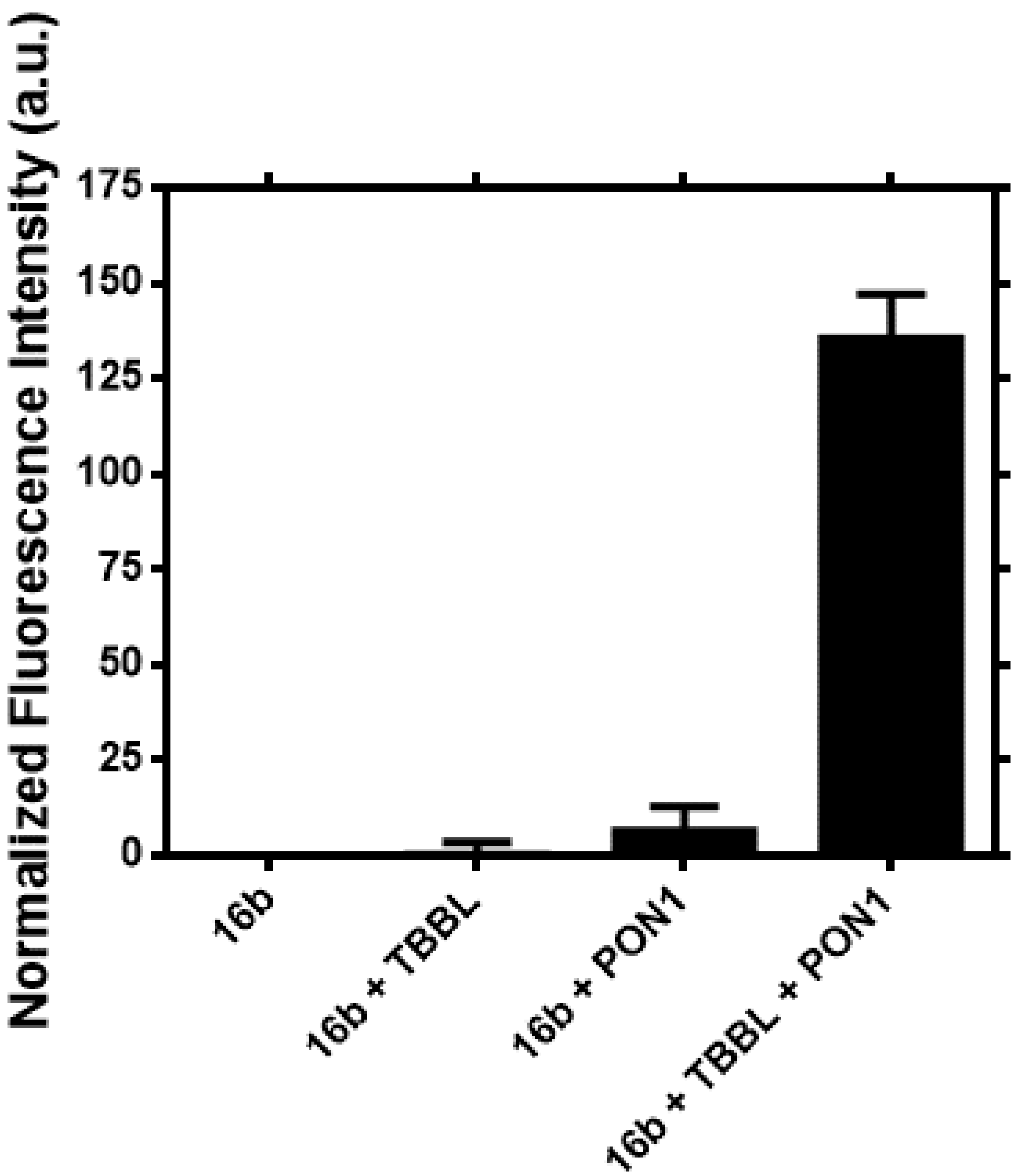

Figure S4. Presence of PON1 and TBBL is required for the fluorogenic property of **16b**. PON1 lactonase activity was determined by measuring the 6-FAM fluorescence released from **16b** in the spectrofluorometer. A **16b** (0.8 μM in DMSO, 1.6%) solution might be supplemented with PON1 (125.6 U L^{-1}, 3.25% glycerol), TBBL (10 mM in ACN, 1%), or both in the Tris buffer. The **16b** reactions were carried out at 25˚C for 30 min and immediately determined fluorescence emission afterward. The normalized fluorescence intensity data at 525 nm were acquired by subtracting a background fluorescence of **16b** at the same wavelength from the original fluorescence intensity readings. Each reaction was analyzed three times in order to acquire the averaged fluorescence values and standard deviation (the error bar).

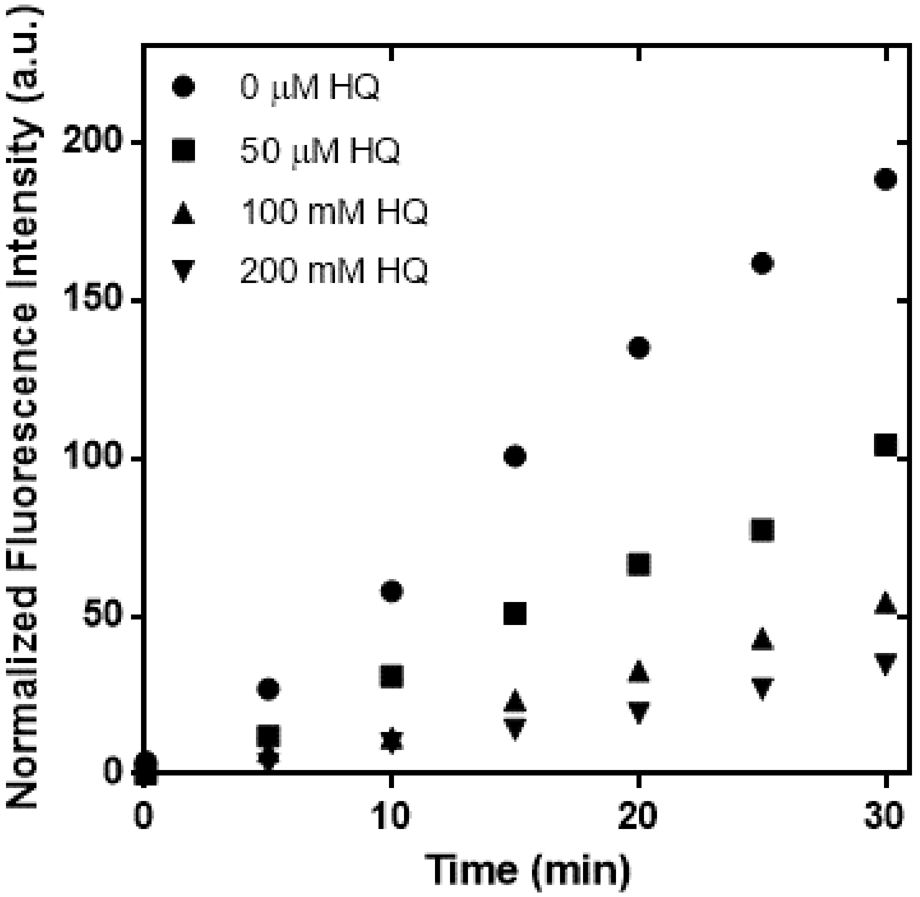

Figure S5. 2-Hydroxyquinoline (HQ) inhibition on PON1 catalysis analyzed by the fluorescence assay based on **16b**. Time-course kinetic analysis of HQ inhibition on PON1 (250.9 U L^{-1}, 1.25% glycerol) catalysis was performed in the presence of TBBL (10 mM in ACN, 1.5%) in the Tris buffer. Each PON1 reaction contained 0, 50, 100 or 200 μM of HQ in 1.5% ACN.

References

[1] J. Dommerholt, S. Schmidt, R. Temming, L.J.A. Hendriks, F.P.J.T. Rutjes, J.C.M. van Hest, D.J. Lefeber, P. Friedl, F.L. van Delft, Readily Accessible Bicyclononynes for Bioorthogonal Labeling and Three-Dimensional Imaging of Living Cells, Angew. Chem., Int. Ed., 49 (2010) 9422-9425.

[2] M.-M. Gong, C.-Y. Dai, S. Severance, C.-C. Hwang, B.-K. Fang, H.-B. Lin, C.-H. Huang, C.-W. Ong, J.-J. Wang, P.-L. Lee, T.-P. Wang, A Bioorthogonally Synthesized and Disulfide-Containing Fluorescence Turn-On Chemical Probe for Measurements of Butyrylcholinesterase Activity and Inhibition in the Presence of Physiological Glutathione, Catalysts, 10 (2020) 1169.

Spectra

Mass Spectrum SmartFormula Report

Analysis Info

Analysis Name	D:\Data\c5\N3RHODAMINE_000005.d	6/4/2018 3:17:54 PM
Method	broadband first signal	Operator: YU HSIAO-CHING
Sample Name	N3-Rhodamine	Instrument: BRUKER FT-MS solariX
Comment	ESI Positive	

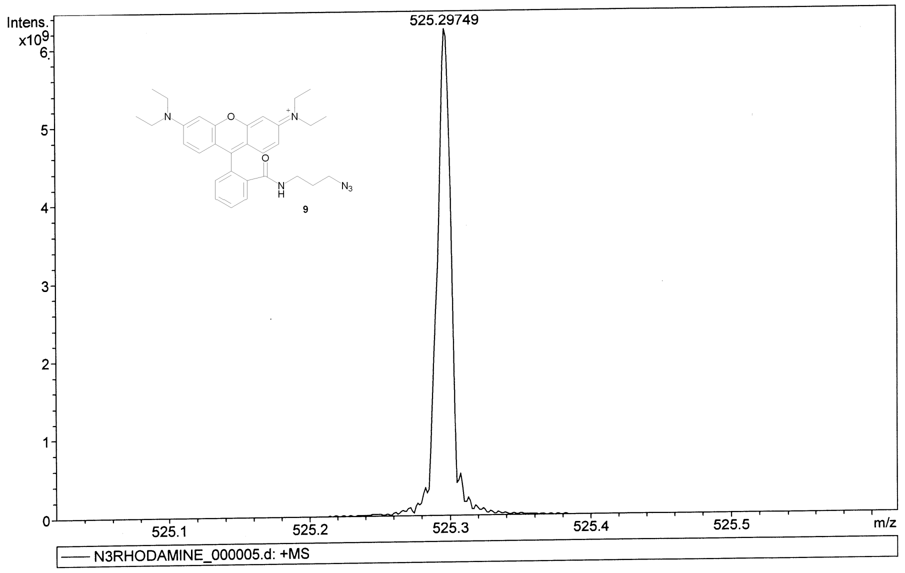

Meas. m/z	#	Formula	Score	m/z	err [mDa]	err [ppm]	mSigma	rdb	e⁻ Conf	N-Rule
525.29749	1	C 31 H 37 N 6 O 2	100.00	525.29725	-0.24	-0.45	8.9	16.5	even	ok

Bruker Compass DataAnalysis 4.0 printed: 6/4/2018 3:17:54 PM Page 1 of 1

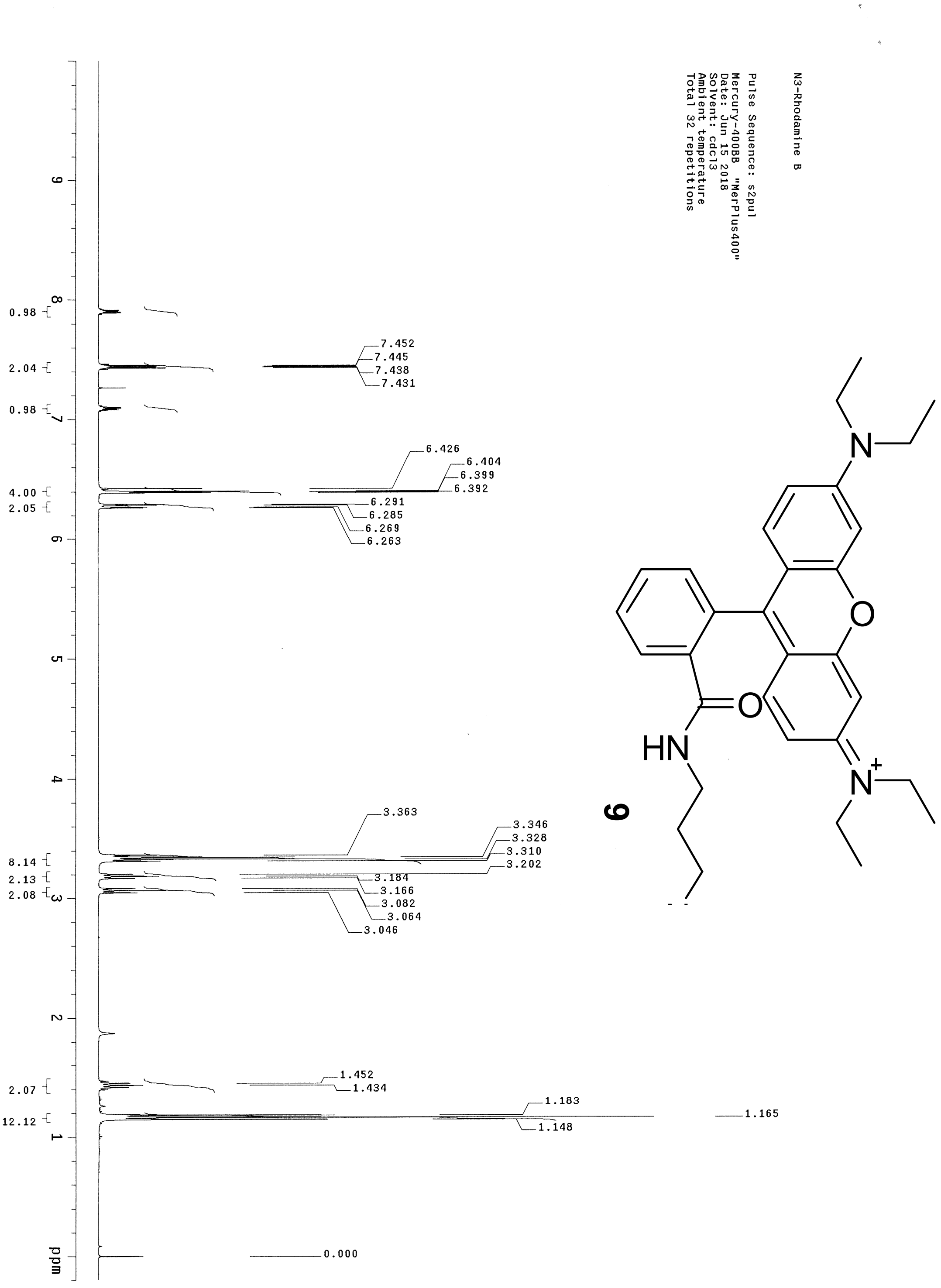
N3-Rhodamine B
Pulse Sequence: s2pul
Mercury-400BB "MerPlus400"
Date: Jun 15 2018
Solvent: cdcl3
Ambient temperature
Total 32 repetitions
7.452
7.445
7.438
7.431
6.426
6.404
6.399
6.392
6.291
6.285
6.269
6.263
3.363
3.346
3.328
3.310
3.202
3.184
3.166
3.082
3.064
3.046
1.452
1.434
1.183
1.165
1.148
0.000
0.98
2.04
0.98
4.00
2.05
8.14
2.13
2.08
2.07
12.12
ppm
N
O
O
HN
N+

9

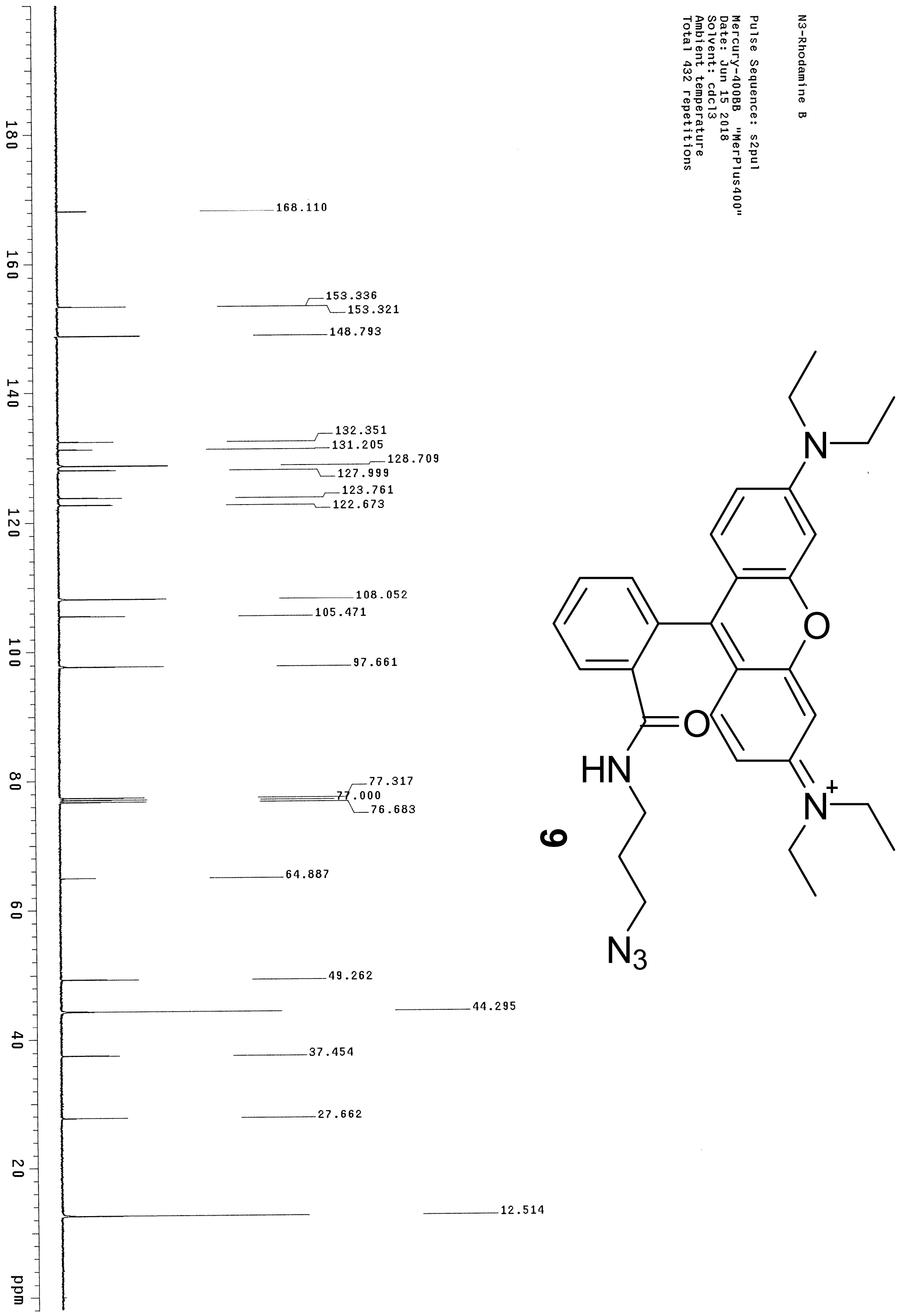
N3-Rhodamine B
Pulse Sequence: s2pul
Mercury-400BB "MerPlus400"
Date: Jun 15 2018
Solvent: cdcl3
Ambient temperature
Total 432 repetitions
168.110
153.336
153.321
148.793
132.351
131.205
128.709
127.999
123.761
122.673
108.052
105.471
97.661
77.317
77.000
76.683
64.887
49.262
44.295
37.454
27.662
12.514
ppm
HN
O
N3
N+
N
9

Mass Spectrum SmartFormula Report

Analysis Info

Analysis Name D:\Data\a4\5CARBOXYFLUORESCEIN_000004.d
Method broadband first signal
Sample Name 5-carboxyfluorescein
Comment ESI Positive

12/31/2020 2:42:36 PM
Operator: YU HSIAO-CHING
Instrument: BRUKER FT-MS solariX

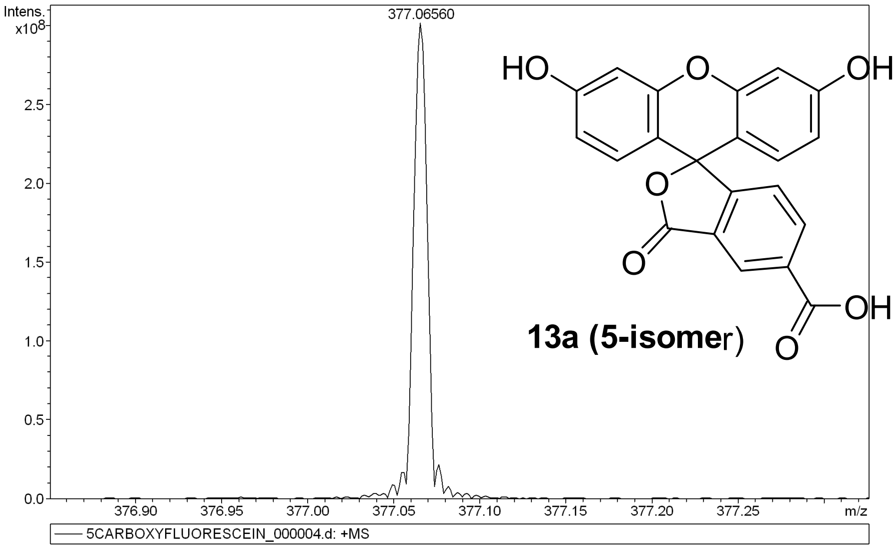

13a (5-isomer)

Meas. m/z	#	Formula	Score	m/z	err [mDa]	err [ppm]	mSigma	rdb	e⁻ Conf	N-Rule
377.06560	1	C 21 H 13 O 7	100.00	377.06558	-0.02	-0.05	8.8	15.5	even	ok

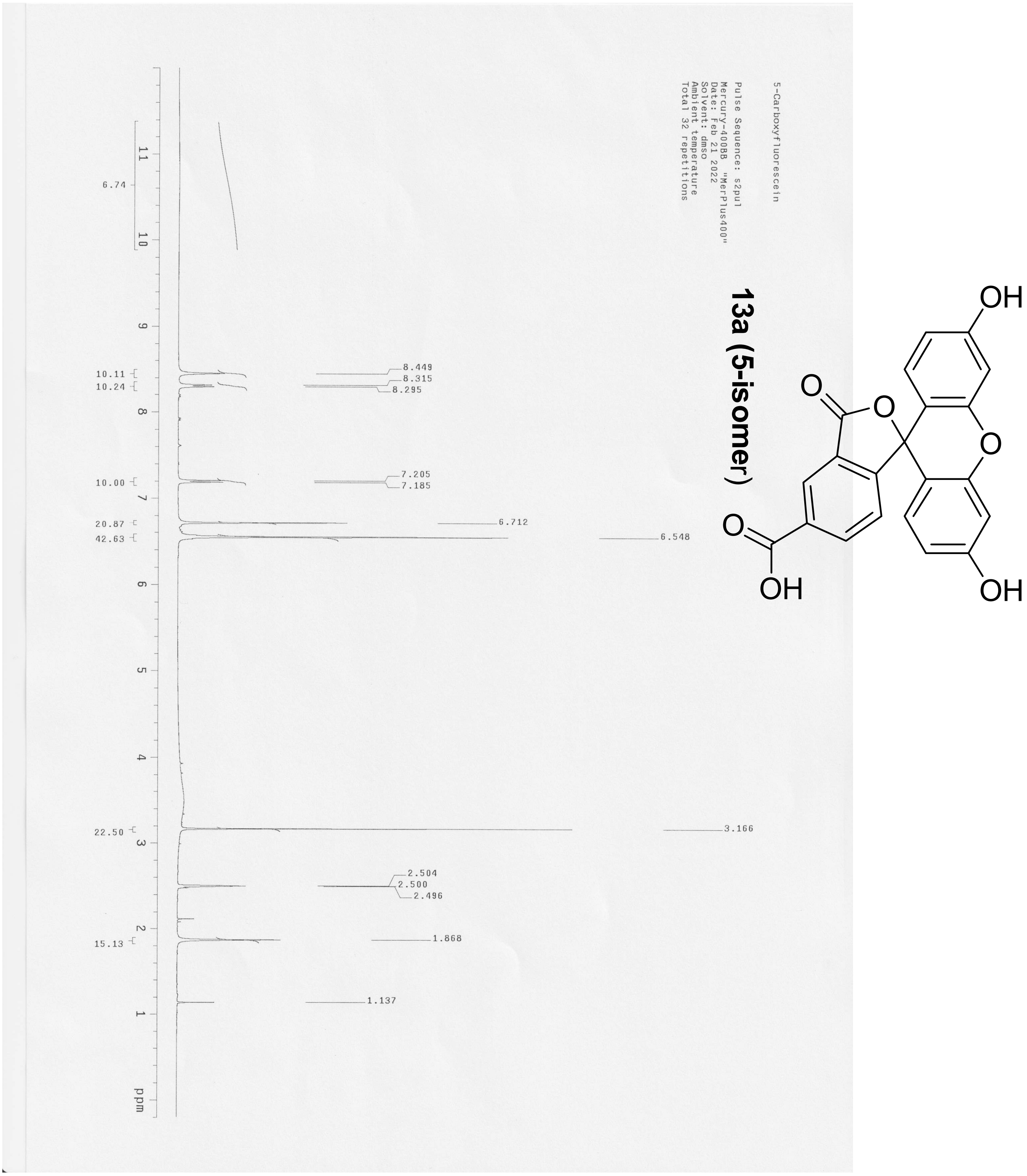
5-Carboxyfluorescein
Pulse Sequence: s2pul
Mercury-400BB "MerPlus400"
Date: Feb 21 2022
Solvent: dmso
Ambient temperature
Total 32 repetitions
13a (5-isomer)
8.449
8.315
8.295
7.205
7.185
6.712
6.548
3.166
2.504
2.500
2.496
1.868
1.137
6.74
10.11
10.24
10.00
20.87
42.63
22.50
15.13
ppm

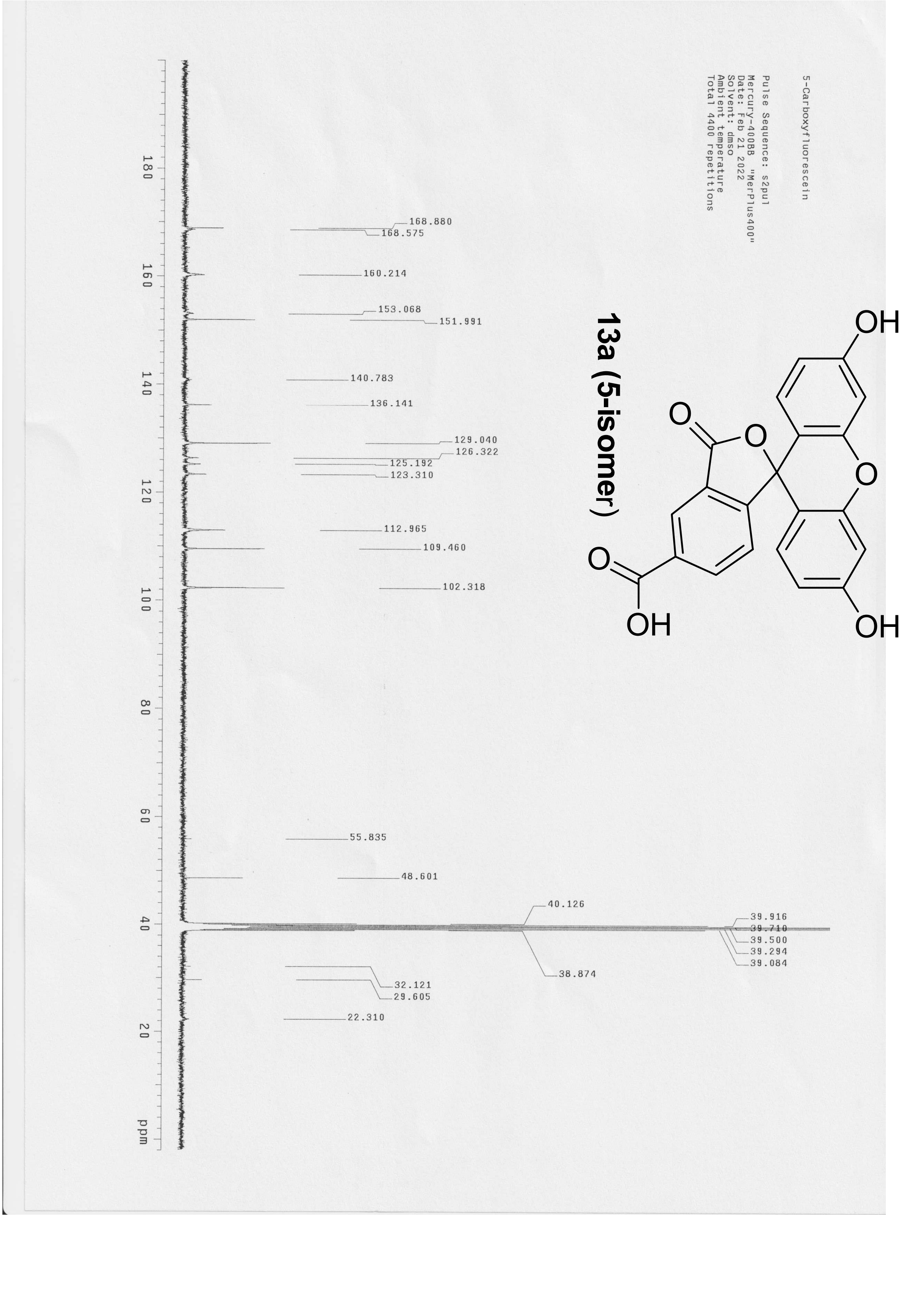
5-Carboxyfluorescein
Pulse Sequence: s2pul
Mercury-400BB "MerPlus400"
Date: Feb 21 2022
Solvent: dmso
Ambient temperature
Total 4400 repetitions
13a (5-isomer)
OH
O
O
O
O
OH
OH
168.880
168.575
160.214
153.068
151.991
140.783
136.141
129.040
126.322
125.192
123.310
112.965
109.460
102.318
55.835
48.601
40.126
39.916
39.710
39.500
39.294
39.084
38.874
32.121
29.605
22.310
180
160
140
120
100
80
60
40
20
ppm

Mass Spectrum SmartFormula Report

Analysis Info

Analysis Name	D:\Data\a4\6CARBOXYFLUORESCEIN_000002.d	12/31/2020 2:44:40 PM
Method	broadband first signal	Operator: YU HSIAO-CHING
Sample Name	6-carboxyfluorescein	Instrument: BRUKER FT-MS solariX
Comment	ESI Positive	

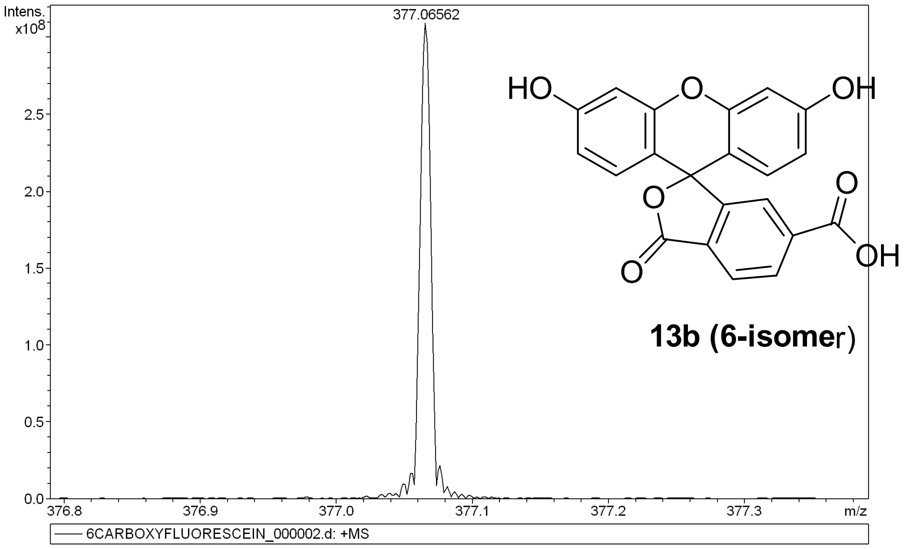

Meas. m/z	#	Formula	Score	m/z	err [mDa]	err [ppm]	mSigma	rdb	e⁻ Conf	N-Rule
377.06562	1	C 21 H 13 O 7	100.00	377.06558	-0.04	-0.10	7.5	15.5	even	ok

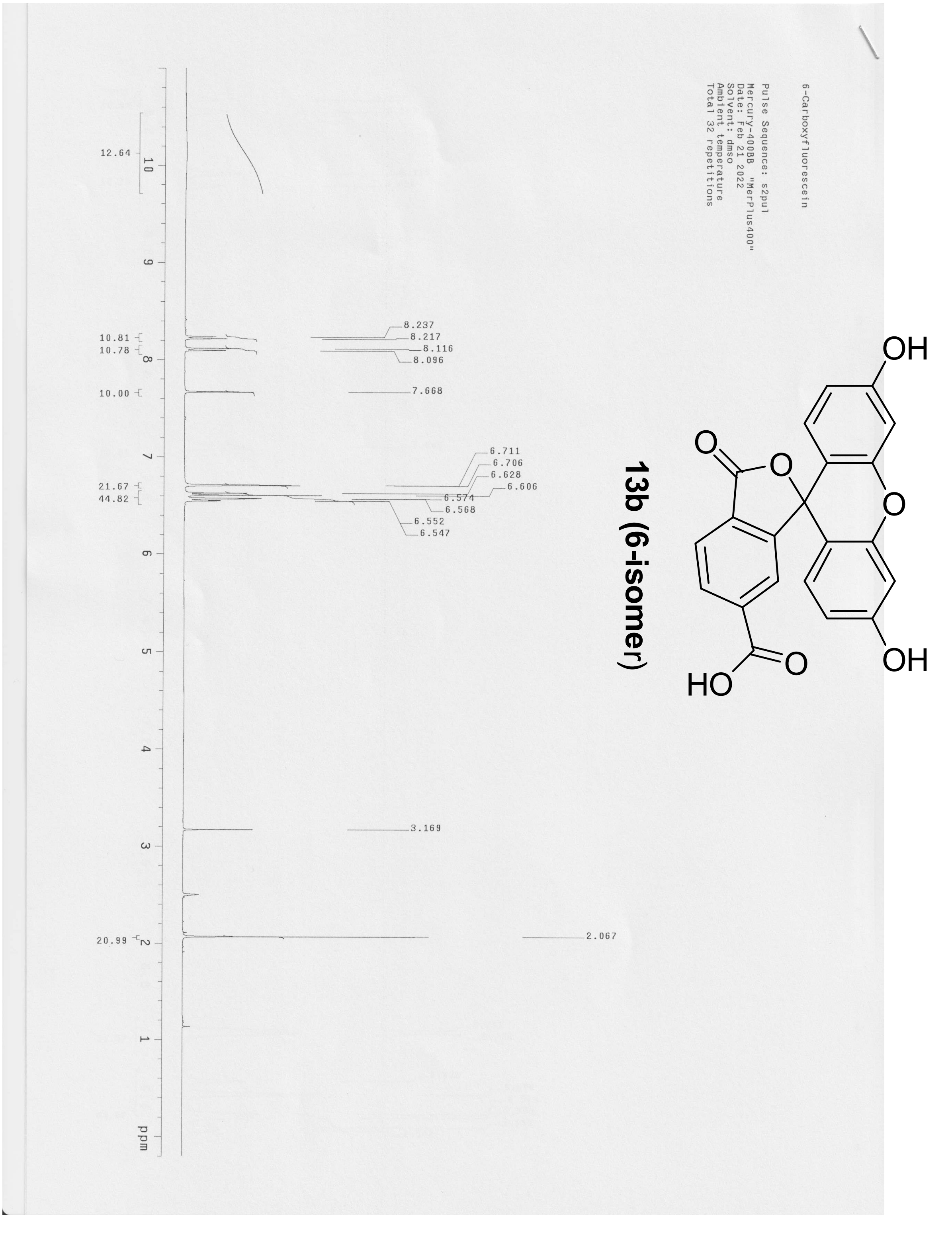
6-Carboxyfluorescein
Pulse Sequence: s2pul
Mercury-400BB "MerPlus400"
Date: Feb 21 2022
Solvent: dmso
Ambient temperature
Total 32 repetitions
13b (6-isomer)
OH
O
HO
ppm

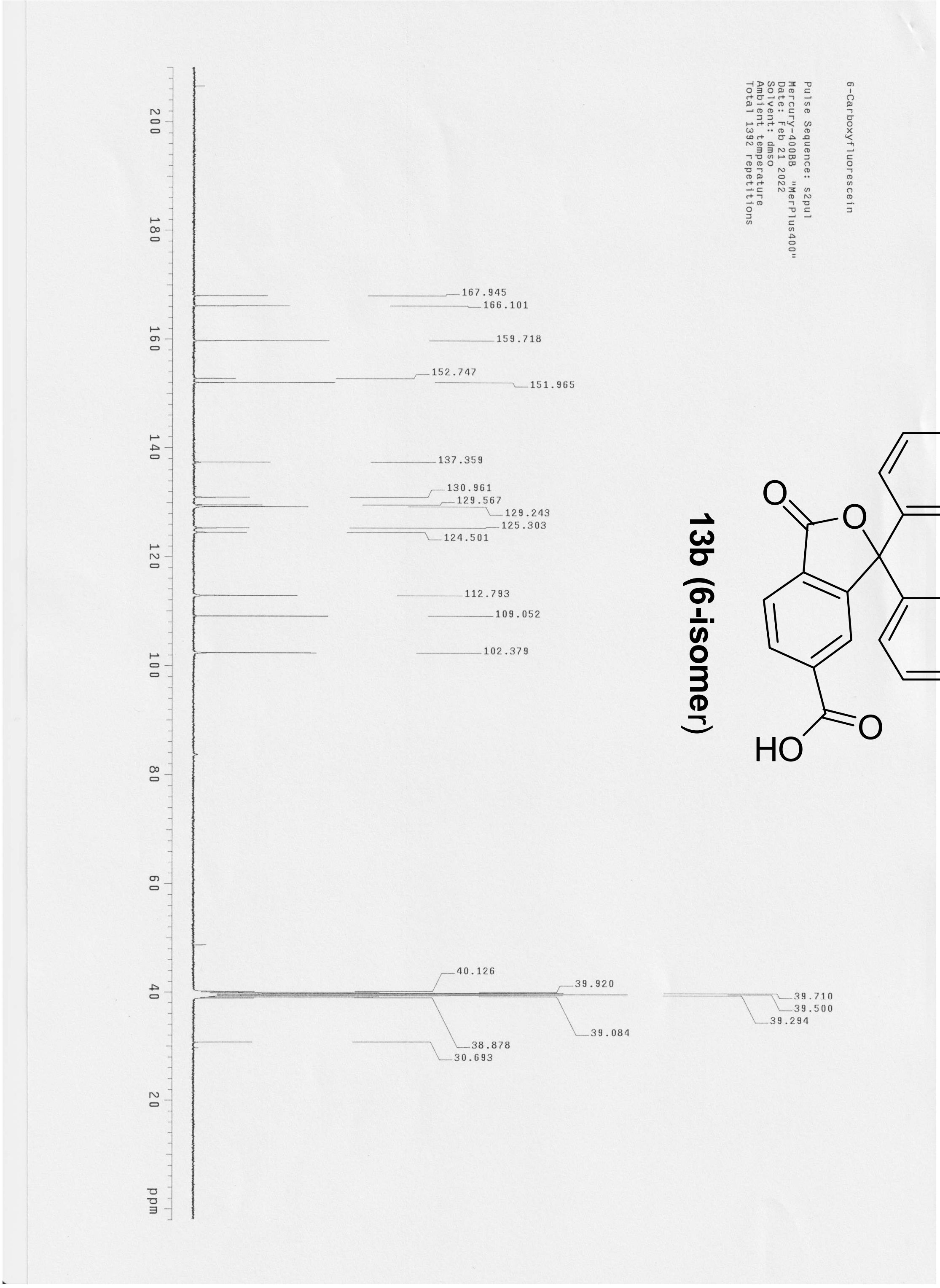

6-Carboxyfluorescein
Pulse Sequence: s2pul
Mercury-400BB "MerPlus400"
Date: Feb 21 2022
Solvent: dmso
Ambient temperature
Total 1392 repetitions
167.945
166.101
159.718
152.747
151.965
137.359
130.961
129.567
129.243
125.303
124.501
112.793
109.052
102.379
40.126
39.920
39.710
39.500
39.294
39.084
38.878
30.693
200
180
160
140
120
100
80
60
40
20
ppm
OH
O
O
O
OH
HO
O
13b (6-isomer)

Mass Spectrum SmartFormula Report

Analysis Info

Analysis Name	D:\Data\a4\5FLUORESCEINNHSESTER_000003.d	12/31/2020 2:55:28 PM
Method	broadband first signal	Operator: YU HSIAO-CHING
Sample Name	5-fluorescein-NHS ester	Instrument: BRUKER FT-MS solariX
Comment	ESI Positive	

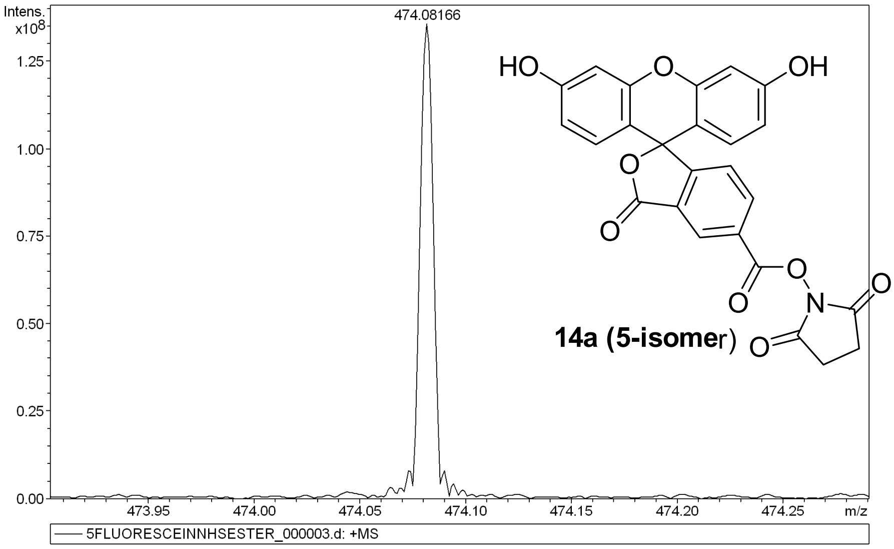

Meas. m/z	#	Formula	Score	m/z	err [mDa]	err [ppm]	mSigma	rdb	e⁻ Conf	N-Rule
474.08166	1	C 25 H 16 N O 9	100.00	474.08196	0.30	0.63	13.1	18.5	even	ok

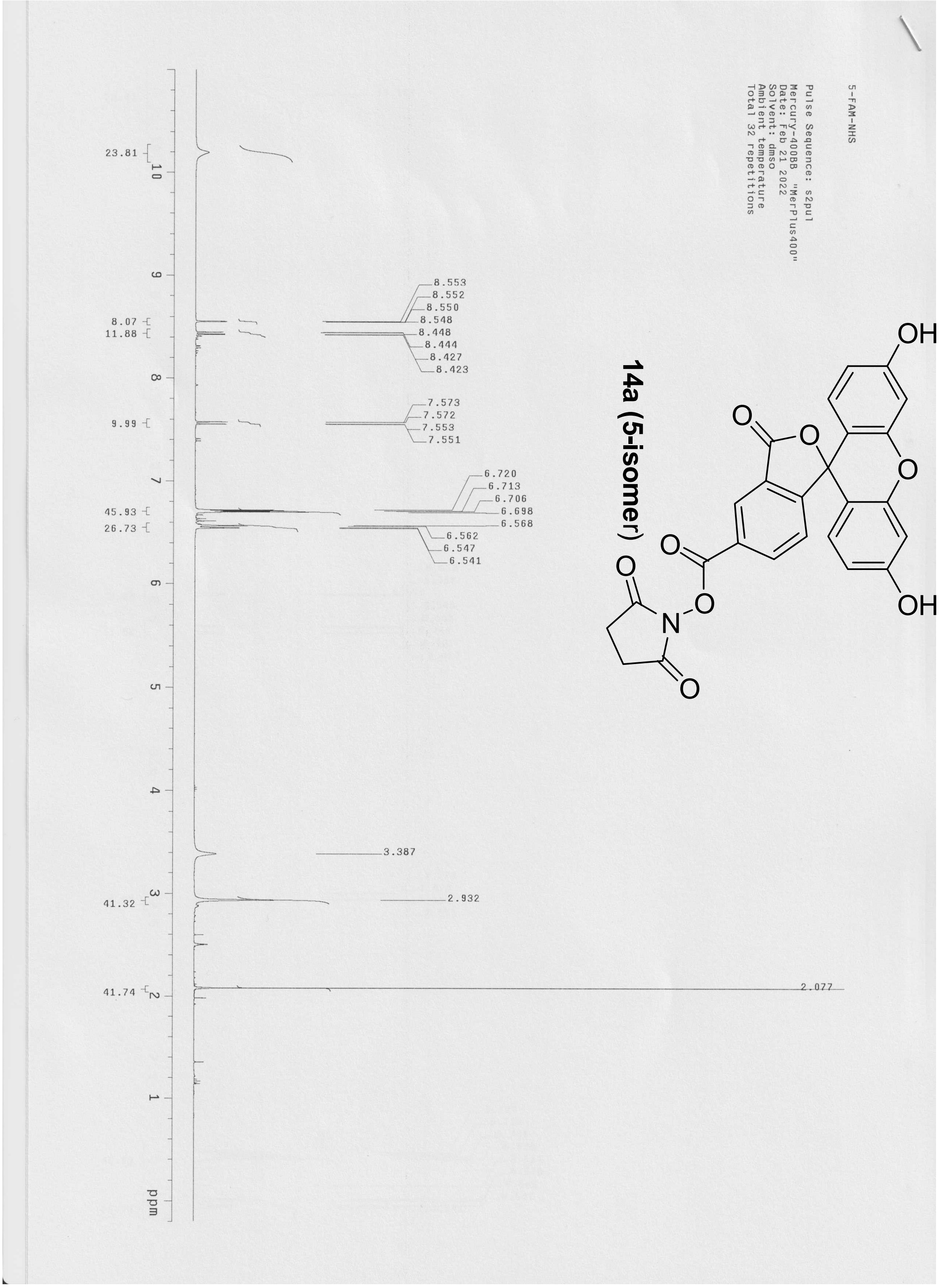
5-FAM-NHS
Pulse Sequence: s2pul
Mercury-400BB "MerPlus400"
Date: Feb 21 2022
Solvent: dmso
Ambient temperature
Total 32 repetitions
14a (5-isomer)
ppm

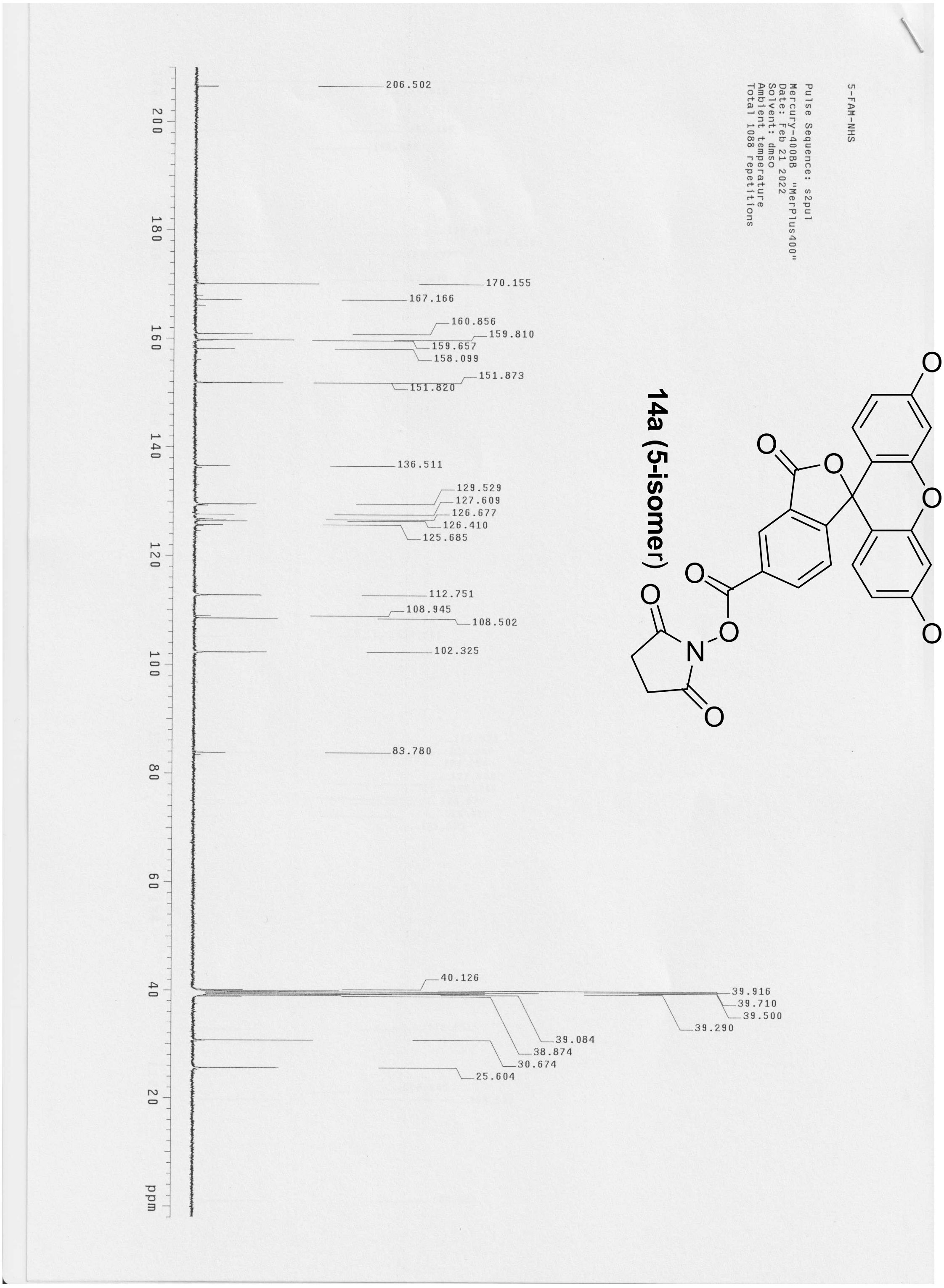
5-FAM-NHS
Pulse Sequence: s2pul
Mercury-400BB "MerPlus400"
Date: Feb 21 2022
Solvent: dmso
Ambient temperature
Total 1088 repetitions
14a (5-isomer)
206.502
170.155
167.166
160.856
159.810
159.657
158.099
151.873
151.820
136.511
129.529
127.609
126.677
126.410
125.685
112.751
108.945
108.502
102.325
83.780
40.126
39.916
39.710
39.500
39.290
39.084
38.874
30.674
25.604
ppm

Mass Spectrum SmartFormula Report

Analysis Info

Analysis Name	D:\Data\a4\6FLUORESCEINNHSESTER_000006.d	12/31/2020 3:15:11 PM
Method	broadband first signal	Operator: YU HSIAO-CHING
Sample Name	6-fluorescein-NHS ester	Instrument: BRUKER FT-MS solariX
Comment	ESI Positive	

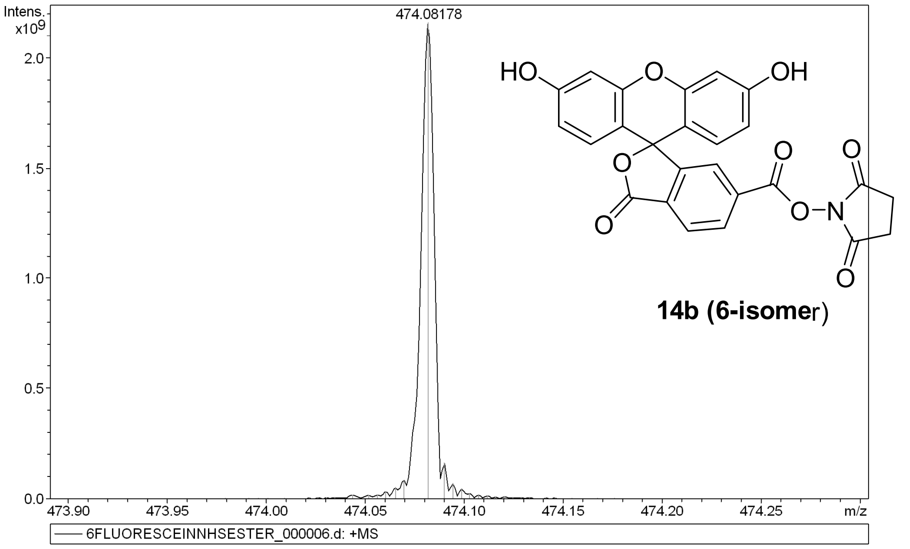

14b (6-isomer)

Meas. m/z	#	Formula	Score	m/z	err [mDa]	err [ppm]	mSigma	rdb	e⁻ Conf	N-Rule
474.08178	1	C 25 H 16 N O 9	100.00	474.08196	0.17	0.37	9.8	18.5	even	ok

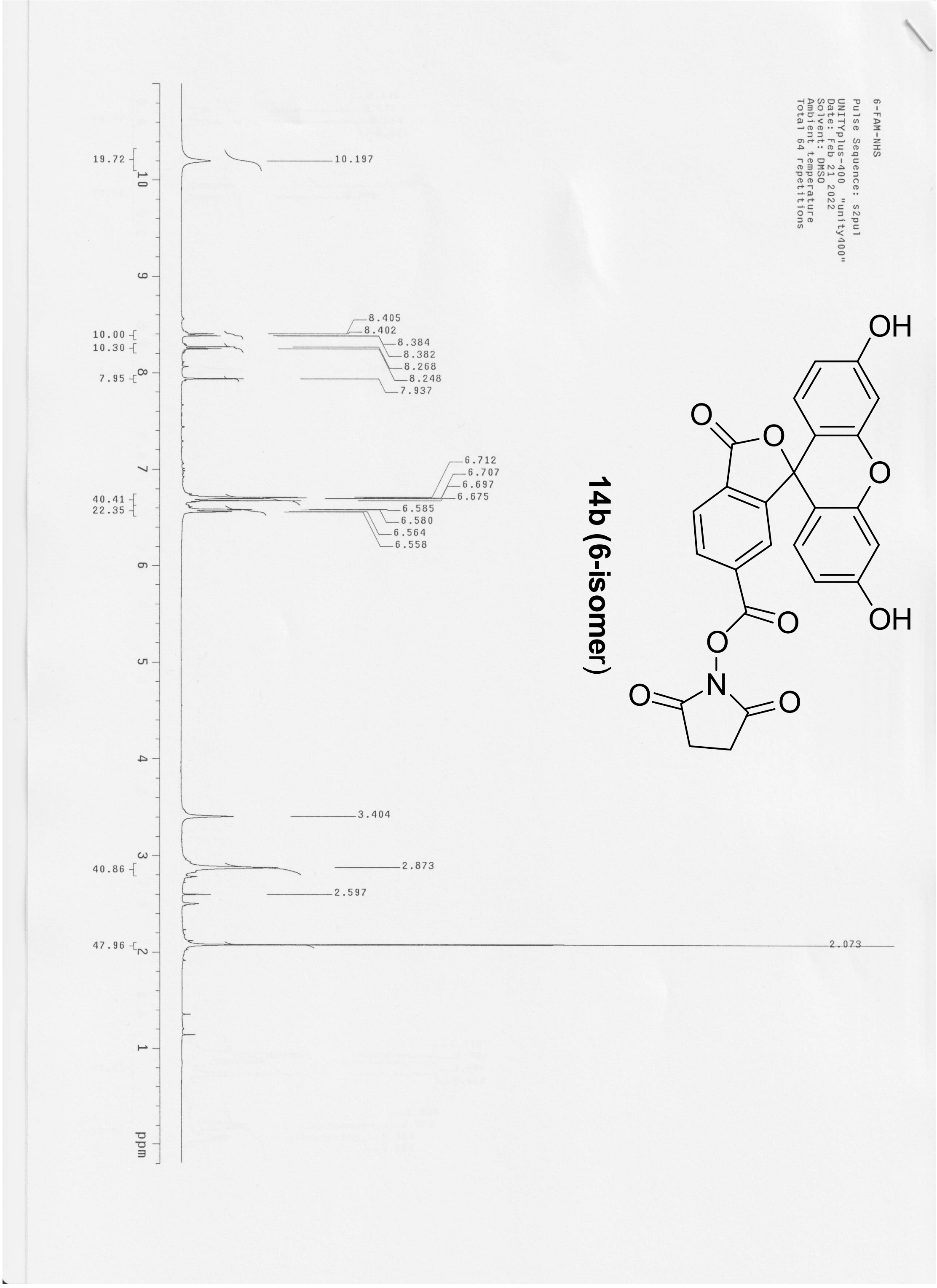
6-FAM-NHS
Pulse Sequence: s2pul
UNITYplus-400 "unity400"
Date: Feb 21 2022
Solvent: DMSO
Ambient temperature
Total 64 repetitions
ppm

14b (6-isomer)

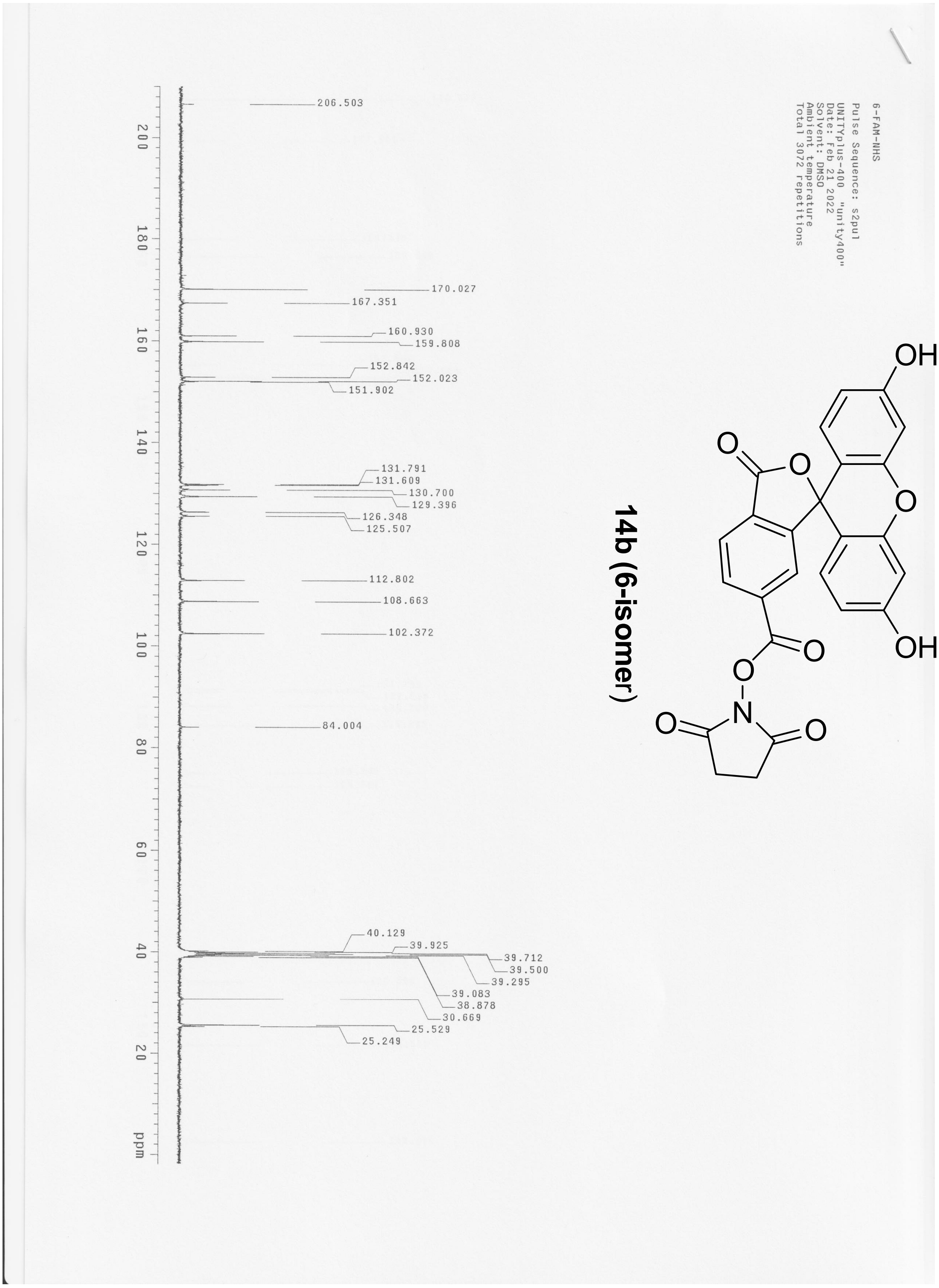
6-FAM-NHS
Pulse Sequence: s2pul
UNITYplus-400 "unity400"
Date: Feb 21 2022
Solvent: DMSO
Ambient temperature
Total 3072 repetitions
206.503
170.027
167.351
160.930
159.808
152.842
152.023
151.902
131.791
131.609
130.700
129.396
126.348
125.507
112.802
108.663
102.372
84.004
40.129
39.925
39.712
39.500
39.295
39.083
38.878
30.669
25.529
25.249
ppm
14b (6-isomer)
OH
O
N

Mass Spectrum SmartFormula Report

Analysis Info

Analysis Name	D:\Data\a4\5FAMEXOBCN5_000002.d	4/9/2021 4:06:24 PM
Method	broadband first signal	Operator: YU HSIAO-CHING
Sample Name	5-FAM-exo-BCN-5	Instrument: BRUKER FT-MS solariX
Comment	ESI Negative	

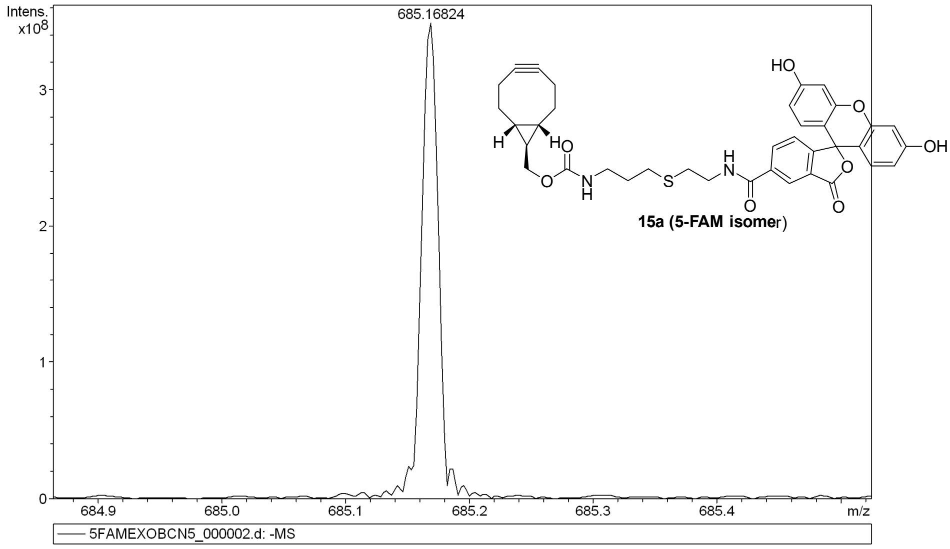

Meas. m/z	#	Formula	Score	m/z	err [mDa]	err [ppm]	mSigma	rdb	e⁻ Conf	N-Rule
685.16824	1	C 36 H 33 N 2 O 8 S 2	100.00	685.16838	0.14	0.20	55.1	21.5	even	ok

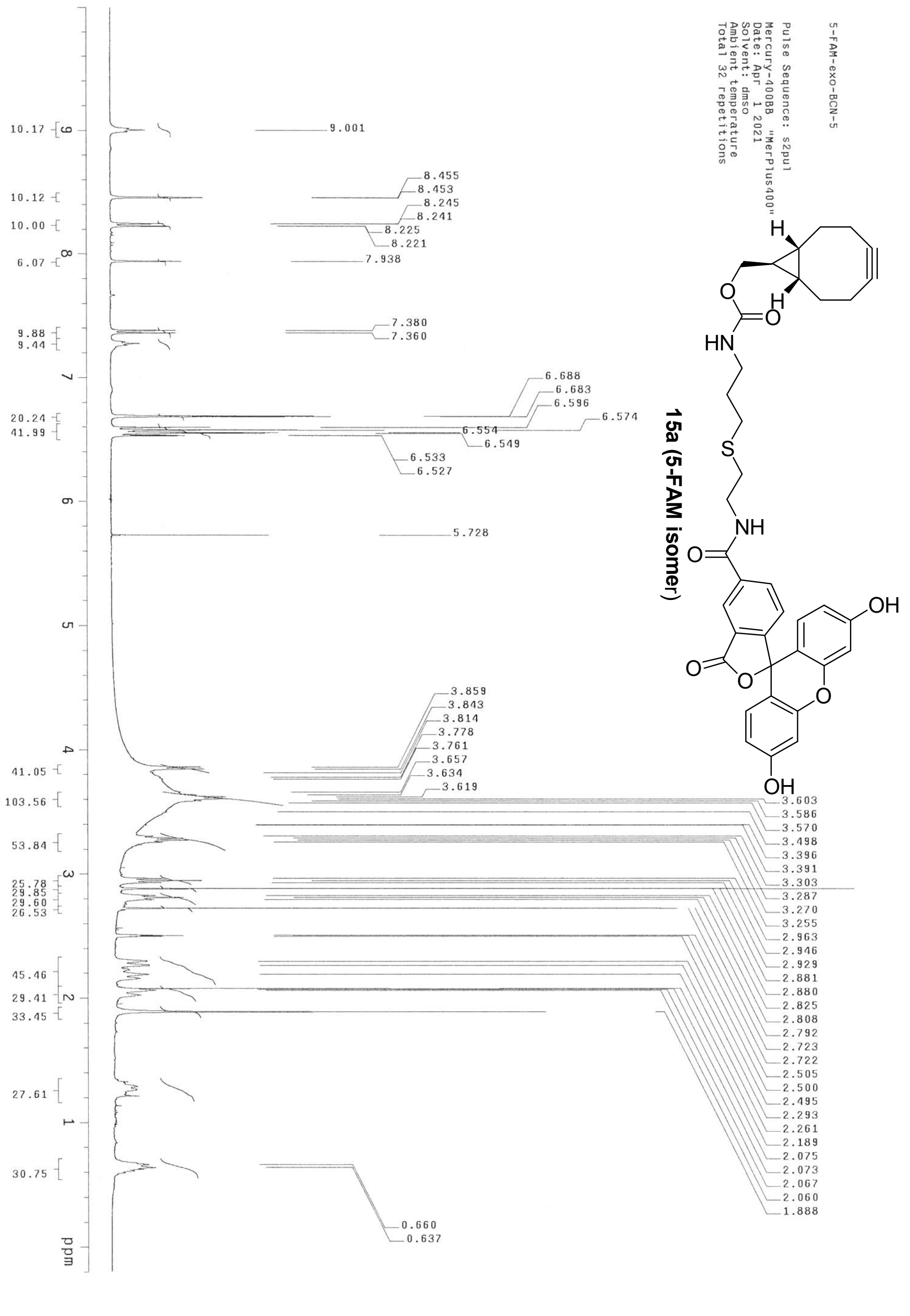

15a (5-FAM isomer)

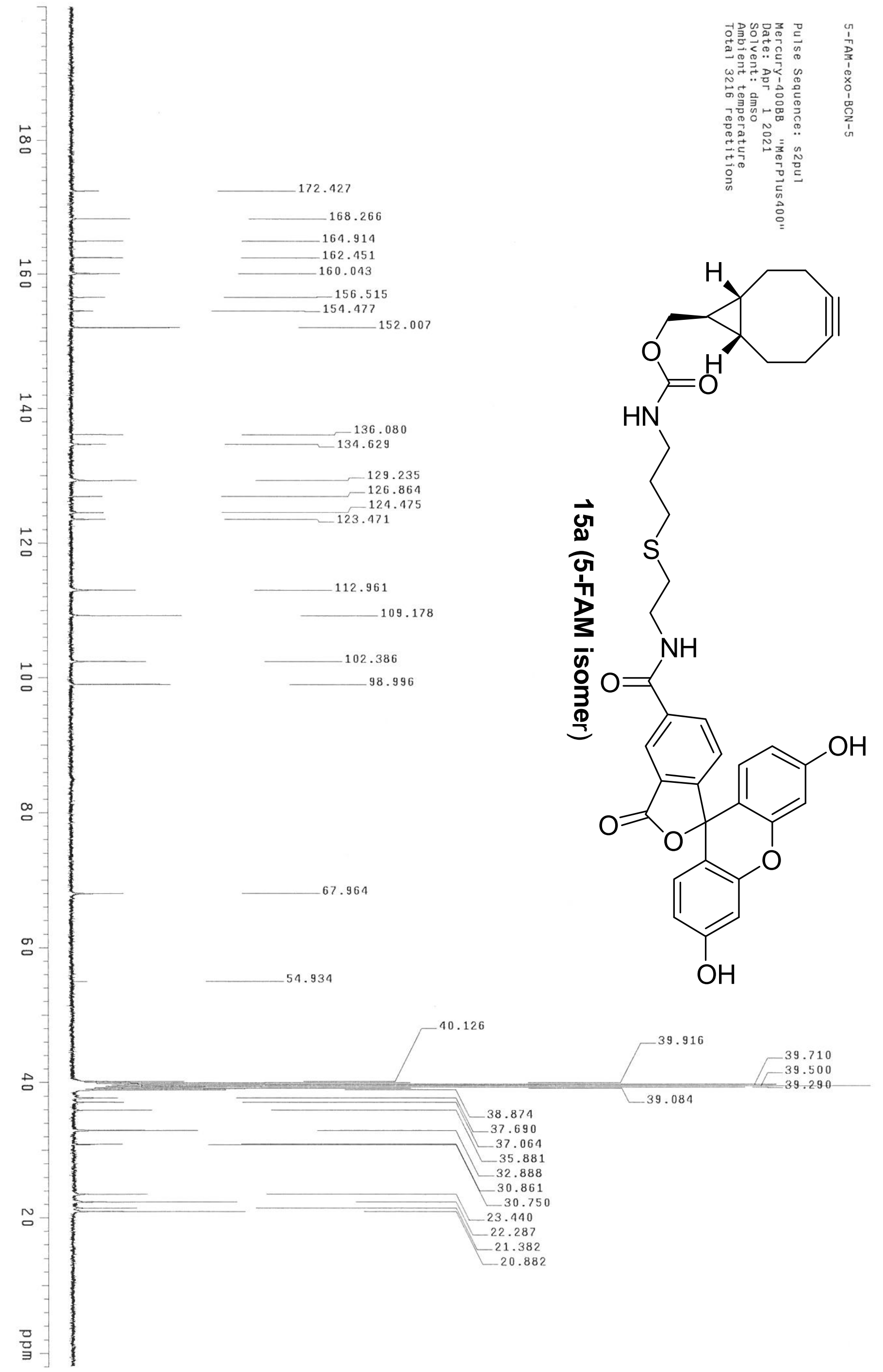
5-FAM-exo-BCN-5
Pulse Sequence: s2pul
Mercury-400BB "MerPlus400"
Date: Apr 1 2021
Solvent: dmso
Ambient temperature
Total 3216 repetitions
15a (5-FAM isomer)
172.427
168.266
164.914
162.451
160.043
156.515
154.477
152.007
136.080
134.629
129.235
126.864
124.475
123.471
112.961
109.178
102.386
98.996
67.964
54.934
40.126
39.916
39.710
39.500
39.290
39.084
38.874
37.690
37.064
35.881
32.888
30.861
30.750
23.440
22.287
21.382
20.882
ppm

Mass Spectrum SmartFormula Report

Analysis Info

Analysis Name	D:\Data\a4\6FAMEXOBCN5_000002.d	4/9/2021 4:20:16 PM
Method	broadband first signal	Operator: YU HSIAO-CHING
Sample Name	6-FAM-exo-BCN-5	Instrument: BRUKER FT-MS solariX
Comment	ESI Negative	

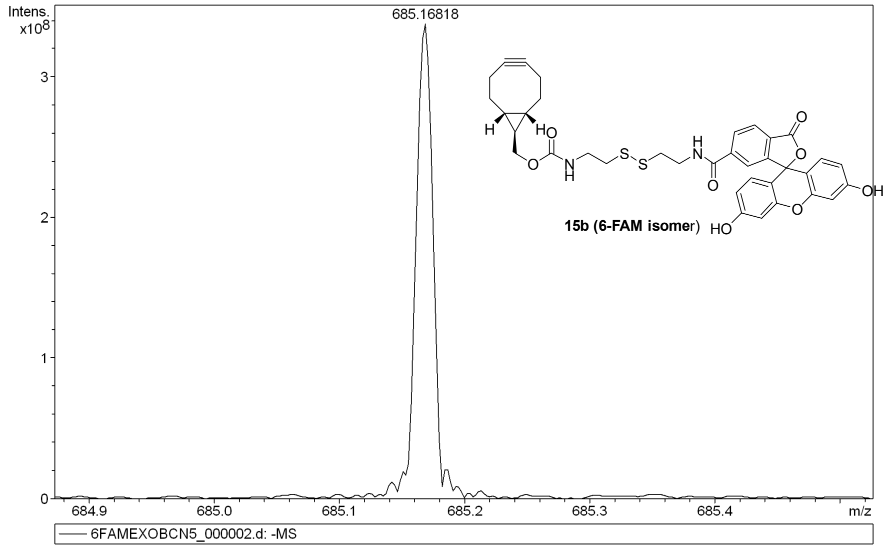

Meas. m/z	#	Formula	Score	m/z	err [mDa]	err [ppm]	mSigma	rdb	e⁻ Conf	N-Rule
685.16818	1	C 36 H 33 N 2 O 8 S 2	100.00	685.16838	0.20	0.29	60.3	21.5	even	ok

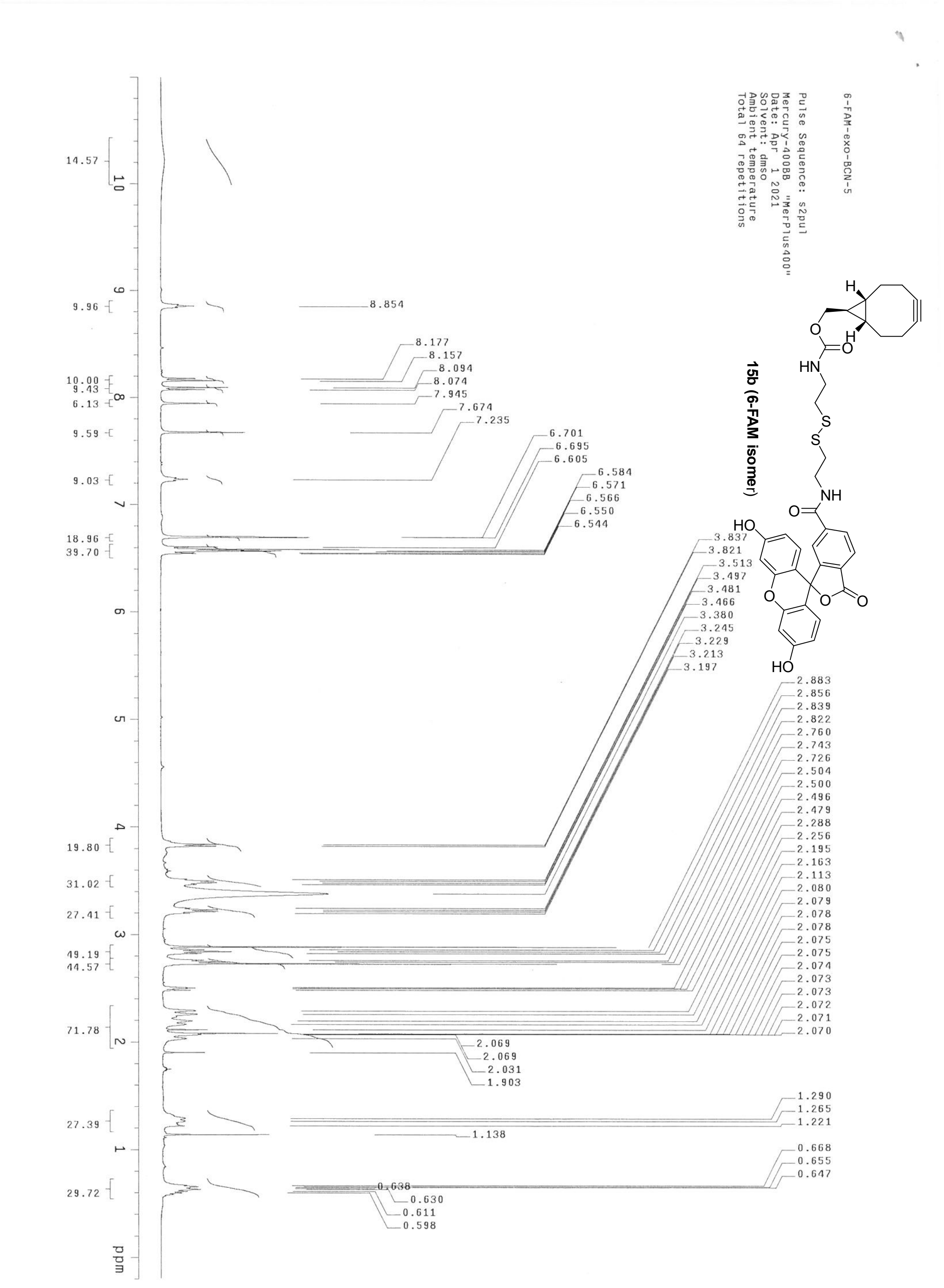
6-FAM-exo-BCN-5
Pulse Sequence: s2pul
Mercury-400BB "MerPlus400"
Date: Apr 1 2021
Solvent: dmso
Ambient temperature
Total 64 repetitions
15b (6-FAM isomer)

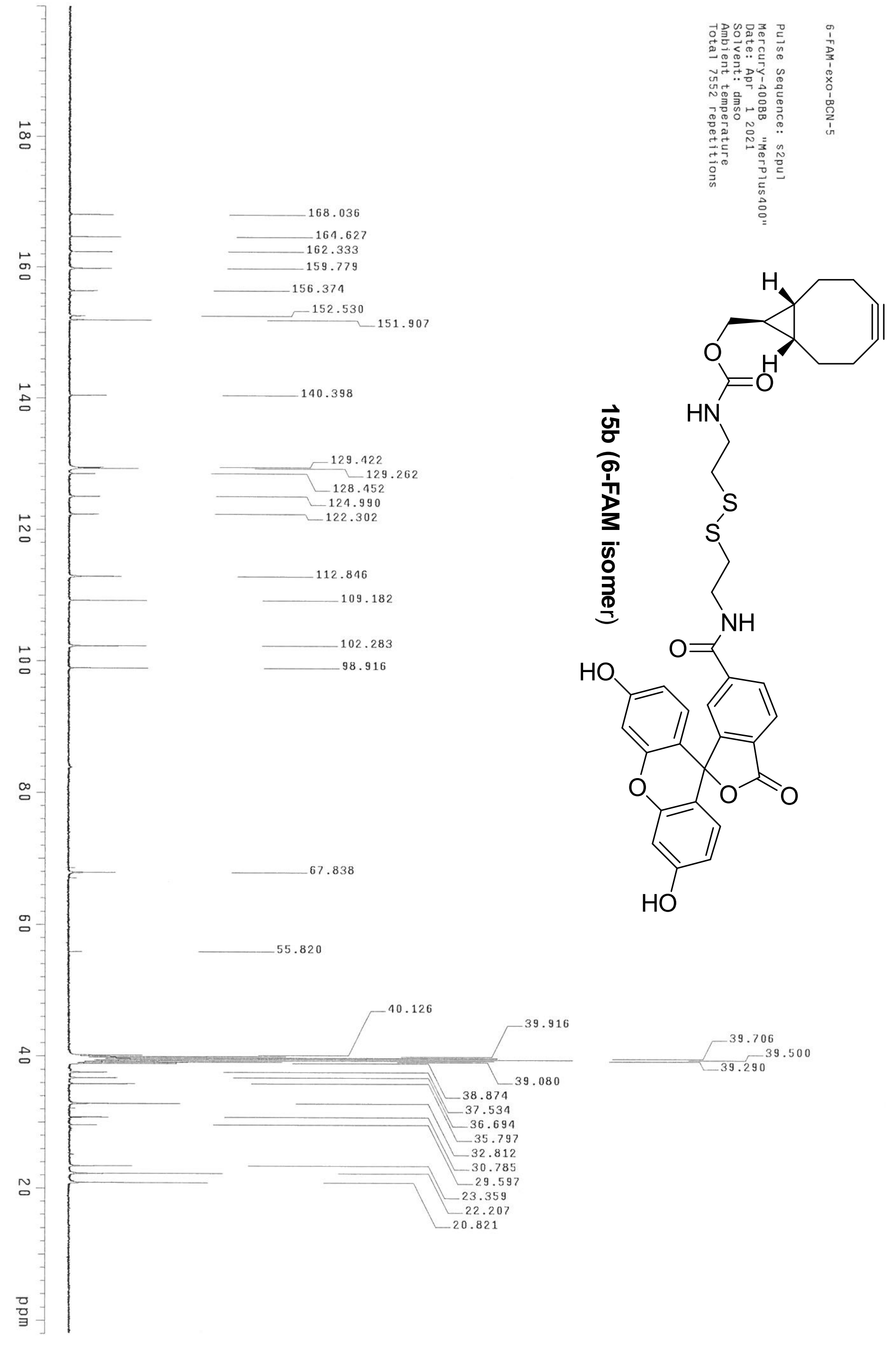
6-FAM-exo-BCN-5
Pulse Sequence: s2pul
Mercury-400BB "MerPlus400"
Date: Apr 1 2021
Solvent: dmso
Ambient temperature
Total 7552 repetitions
15b (6-FAM isomer)
168.036
164.627
162.333
159.779
156.374
152.530
151.907
140.398
129.422
129.262
128.452
124.990
122.302
112.846
109.182
102.283
98.916
67.838
55.820
40.126
39.916
39.706
39.500
39.290
39.080
38.874
37.534
36.694
35.797
32.812
30.785
29.597
23.359
22.207
20.821
ppm

Mass Spectrum SmartFormula Report

Analysis Info

Analysis Name	D:\Data\a4\EXOBCN65_000024.d	12/31/2020 4:17:31 PM
Method	broadband first signal	Operator: YU HSIAO-CHING
Sample Name	exo-BCN5-6(5)	Instrument: BRUKER FT-MS solariX
Comment	ESI Negtive	

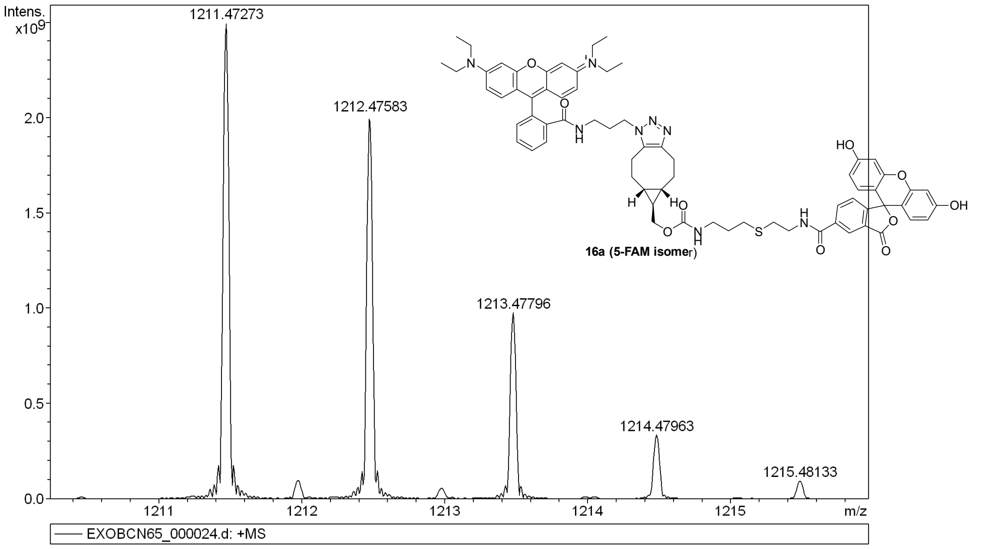

Meas. m/z	#	Formula	Score	m/z	err [mDa]	err [ppm]	mSigma	rdb	e⁻ Conf	N-Rule
1211.47273	1	C 67 H 71 N 8 O 10 S 2	100.00	1211.47291	0.18	0.14	17.8	36.5	even	ok

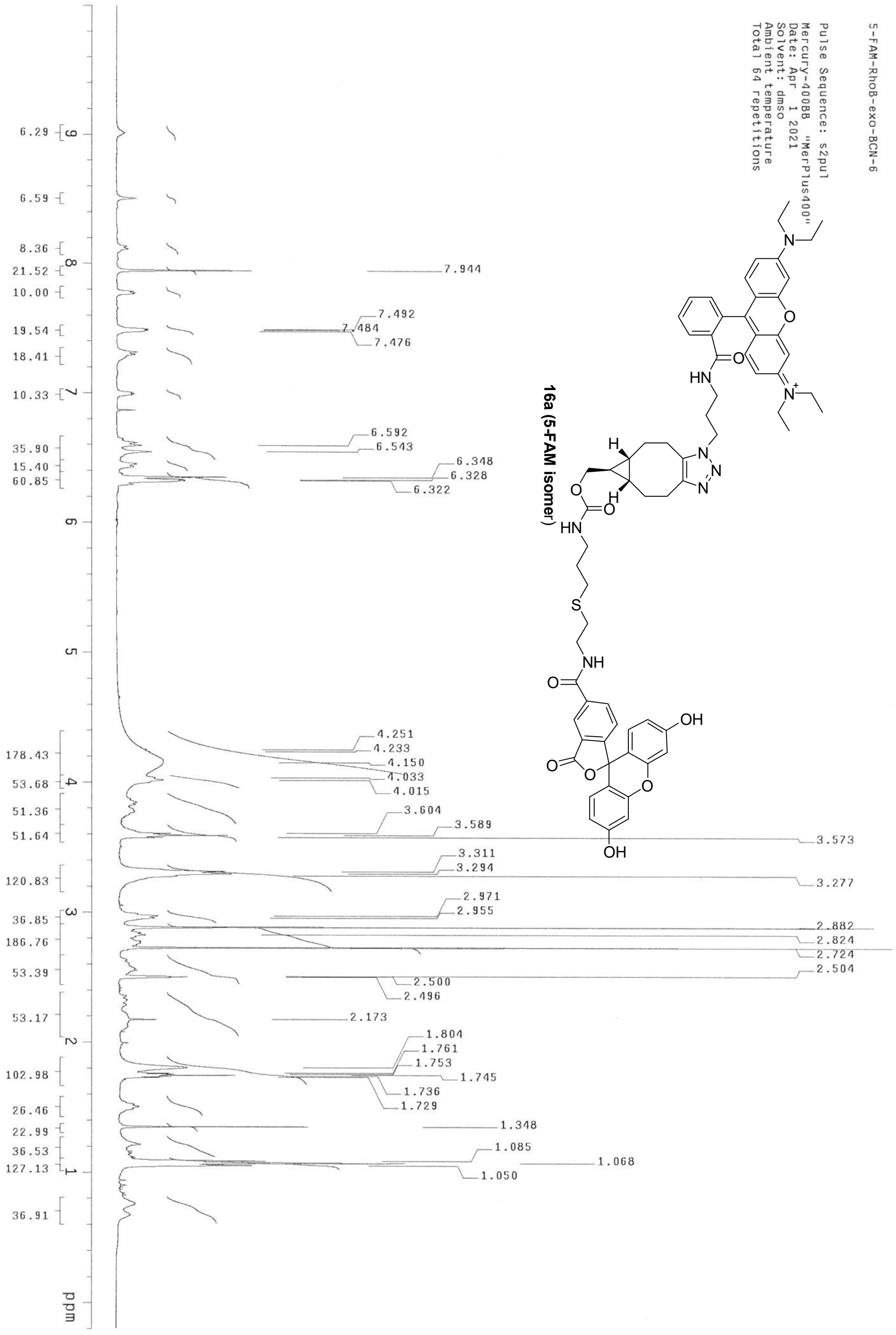
5-FAM-RhoB-exo-BCN-6
Pulse Sequence: s2pul
Mercury-400BB "MerPlus400"
Date: Apr 1 2021
Solvent: dmso
Ambient temperature
Total 64 repetitions
16a (5-FAM isomer)
ppm

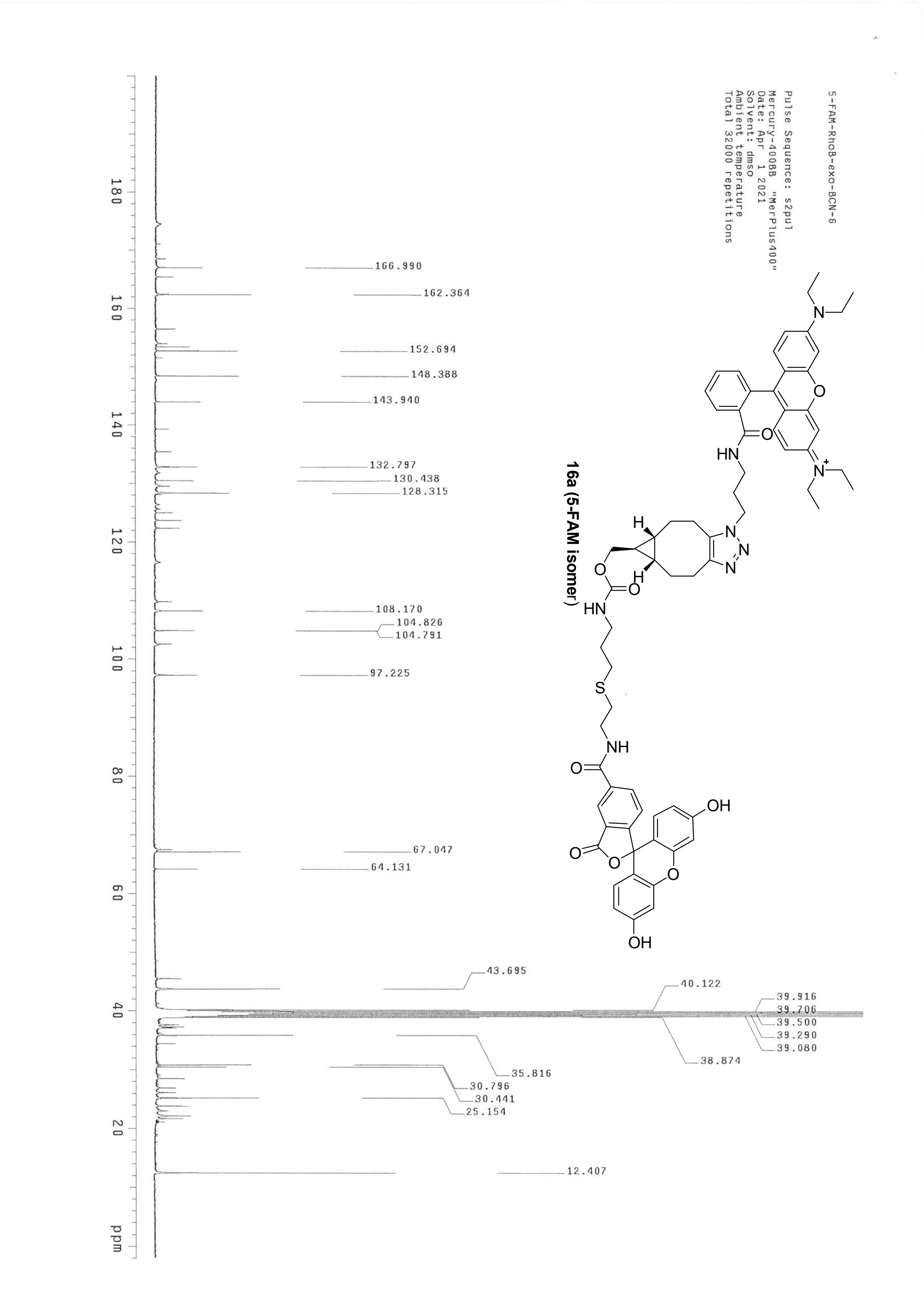
5-FAM-RhoB-exo-BCN-6
Pulse Sequence: s2pul
Mercury-400BB "MerPlus400"
Date: Apr 1 2021
Solvent: dmso
Ambient temperature
Total 3200 repetitions
166.990
162.364
152.694
148.388
143.940
132.797
130.438
128.315
108.170
104.826
104.791
97.225
67.047
64.131
43.695
40.122
39.916
39.706
39.500
39.290
39.080
38.874
35.816
30.796
30.441
25.154
12.407
180
160
140
120
100
80
60
40
20
ppm
16a (5-FAM isomer)

Mass Spectrum SmartFormula Report

Analysis Info

Analysis Name	D:\Data\a4\RHOB6FAMEXOBCN6_000003.d	4/9/2021 3:56:39 PM
Method	broadband first signal	Operator: YU HSIAO-CHING
Sample Name	Rhoab-6-FAM-exo-BCN-6	Instrument: BRUKER FT-MS solariX
Comment	ESI Negative	

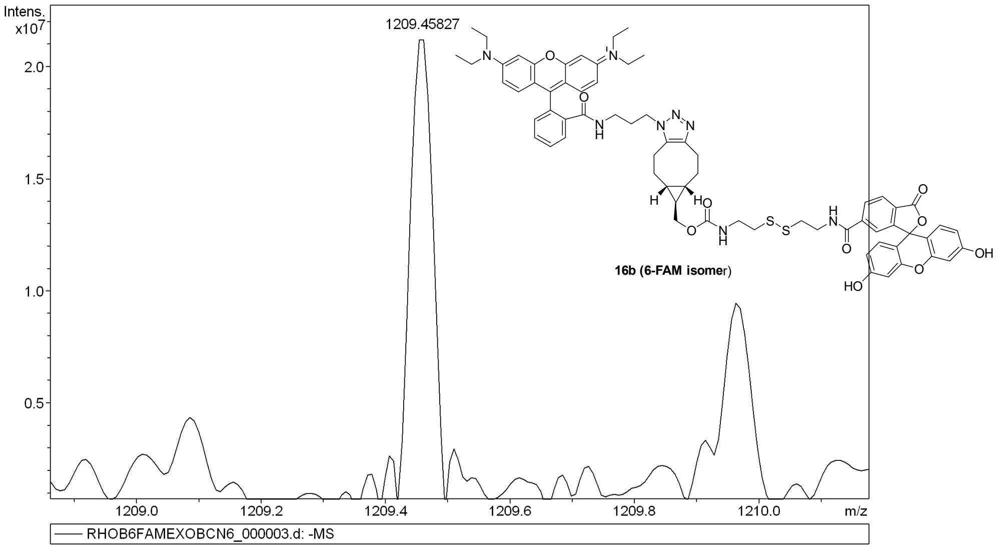

Meas. m/z	#	Formula	Score	m/z	err [mDa]	err [ppm]	mSigma	rdb	e⁻ Conf	N-Rule
1209.45827	1	C 67 H 69 N 8 O 10 S 2	100.00	1209.45836	0.09	0.07	132.2	37.5	even	ok

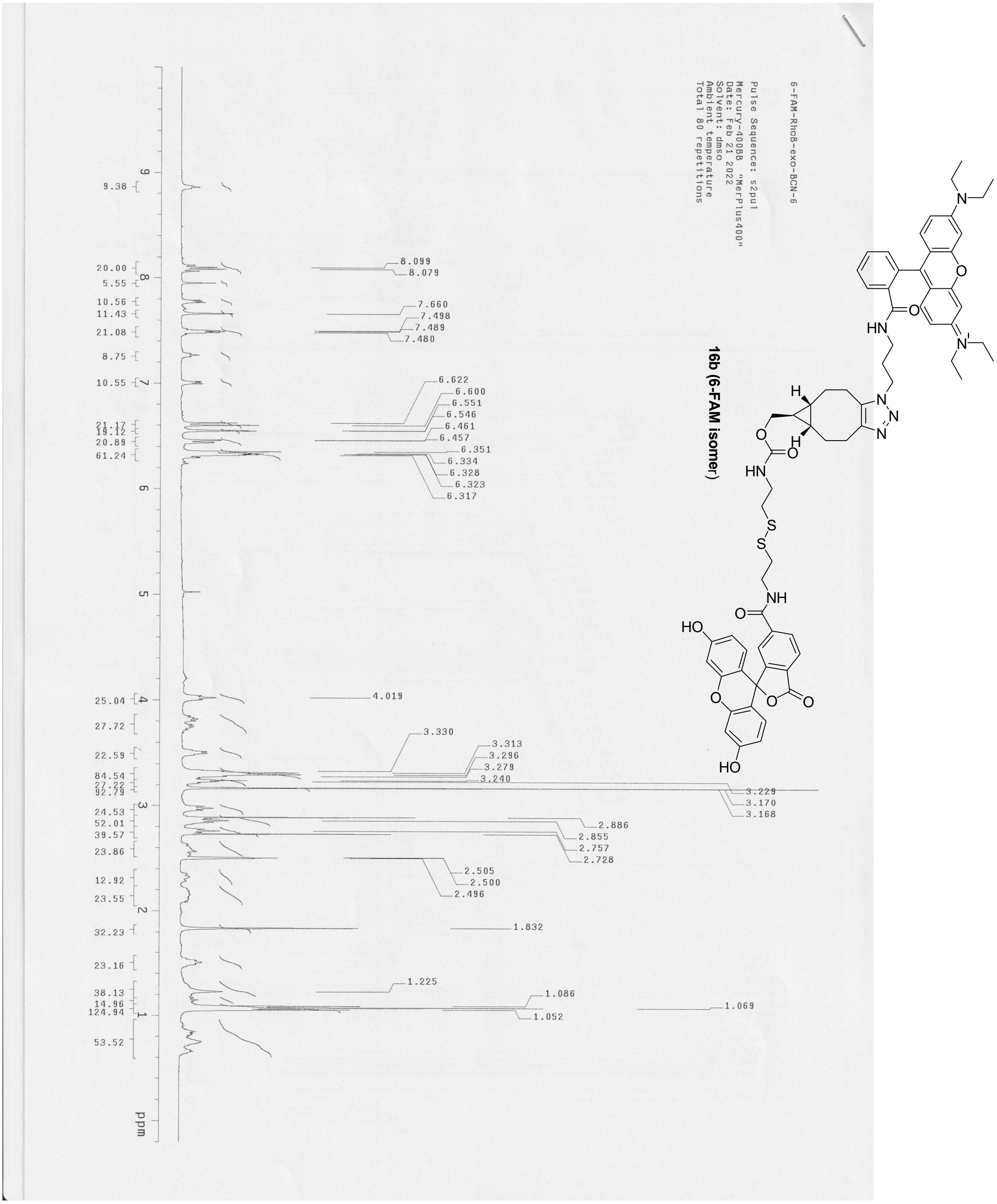
6-FAM-RhoB-exo-BCN-6
Pulse Sequence: s2pul
Mercury-400BB "MerPlus400"
Date: Feb 21 2022
Solvent: dmso
Ambient temperature
Total 80 repetitions
16b (6-FAM isomer)

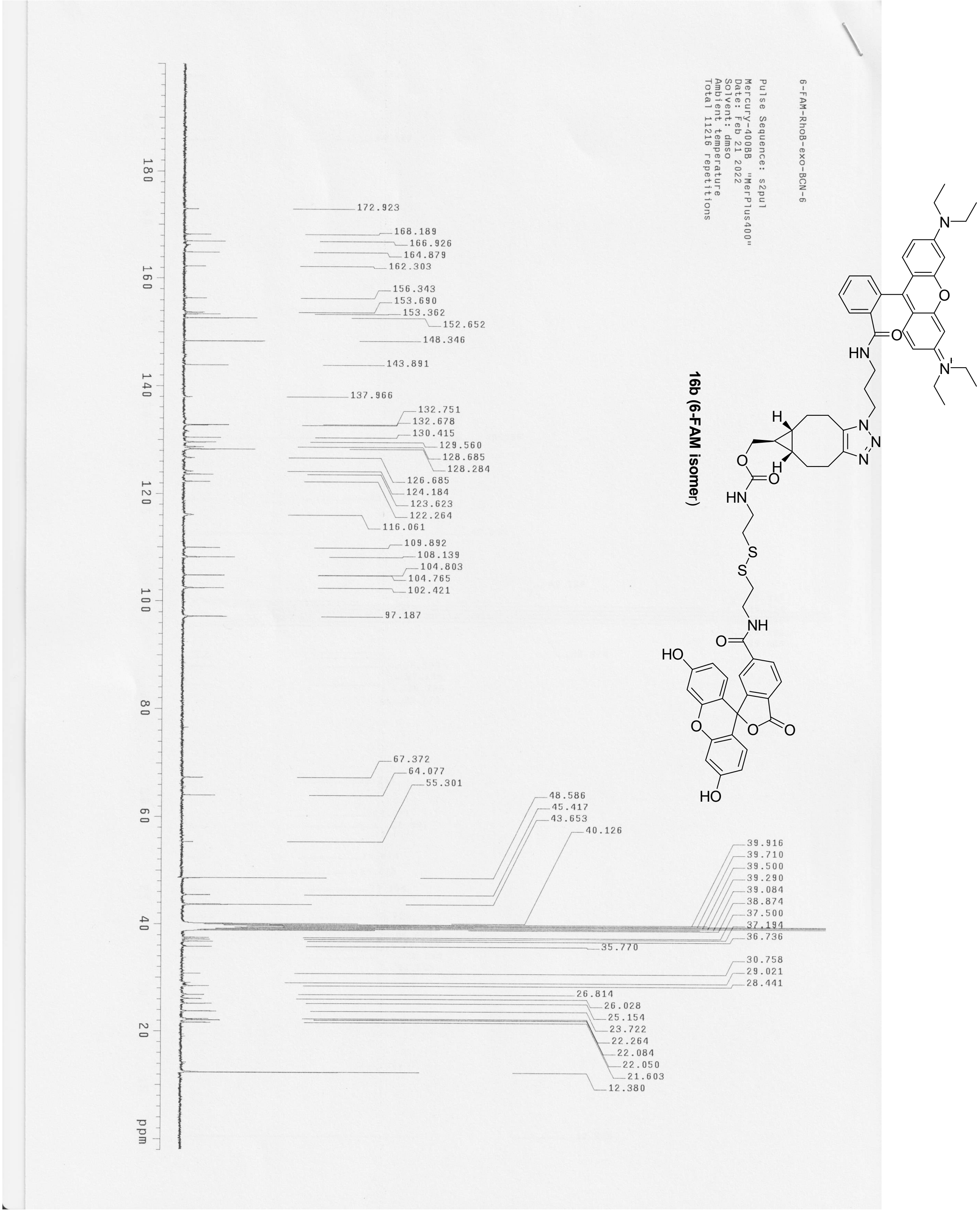
6-FAM-RhoB-exo-BCN-6
Pulse Sequence: s2pul
Mercury-400BB "MerPlus400"
Date: Feb 21 2022
Solvent: dmso
Ambient temperature
Total 1126 repetitions
16b (6-FAM isomer)
172.923
168.189
166.926
164.879
162.303
156.343
153.690
153.362
152.652
148.346
143.891
137.966
132.751
132.678
130.415
129.560
128.685
128.284
126.685
124.184
123.623
122.264
116.061
109.892
108.139
104.803
104.765
102.421
97.187
67.372
64.077
55.301
48.586
45.417
43.653
40.126
39.916
39.710
39.500
39.290
39.084
38.874
37.500
37.194
36.736
35.770
30.758
29.021
28.441
26.814
26.028
25.154
23.722
22.264
22.084
22.050
21.603
12.380
180
160
140
120
100
80
60
40
20
ppm
